# HIT101308137 and HIT104293658 nominate dual target chemotypes for PTPN1 and PTPN2 with preliminary selectivity in colorectal cancer cells

**DOI:** 10.3389/fchem.2026.1782252

**Published:** 2026-04-17

**Authors:** Chaojie Huang, Jingyun Chen, Rui Chen, Liping Cao

**Affiliations:** 1 Department of Colorectal Surgery, Sir Run Run Shaw Hospital, Zhejiang University School of Medicine, Hangzhou, China; 2 School of Bioengineering, Jiangnan University, Wuxi, China; 3 Department of General Surgery, Sir Run Run Shaw Hospital, Zhejiang University School of Medicine, Hangzhou, China

**Keywords:** CCK 8, colorectal cancer, dual target inhibitor, HIT101308137, HIT104293658, molecular dynamics, PTPN1, PTPN2

## Abstract

Colorectal cancer remains a major clinical challenge, and dual targeting of PTPN1 and PTPN2 represents a promising strategy to modulate conserved phosphatase signaling relevant to tumor biology and immune regulation. Here, we identified and prioritized two compounds, HIT101308137 and HIT104293658, as dual compatible chemotypes for PTPN1 and PTPN2 through a structure guided workflow integrating pocket comparison, ligand based interaction hypothesis generation, and structure based evaluation under static and dynamic conditions. Sequence and structural analyses supported high conservation of the catalytic pocket, providing a feasible basis for dual target coverage. Screening and post docking tri metric prioritization nominated three shared candidates, of which HIT101308137 and HIT104293658 were further differentiated by microsecond molecular dynamics simulations and interaction occupancy profiling. In both targets, the candidates preserved an Arg centered anchoring pattern within the P loop, while exhibiting distinct binding behaviors: HIT101308137 maintained a more co crystal like, catalytic core coupled interaction network with stronger persistence of contacts associated with the WPD region, whereas HIT104293658 displayed a more entrance biased interaction distribution consistent with increased pose reorganization. In CCK 8 viability assays, HIT101308137 and HIT104293658 produced reproducible dose dependent reductions in viability in HCT116 and SW480 cells, while showing limited effects in NCM460 and FHC cells within the same concentration window. Together, these results nominate HIT101308137 and HIT104293658 as starting points for dual target optimization and provide a mechanistic rationale to enhance cellular potency while maintaining low impact on normal colonic epithelial cells.

## 1 Introduction

Colorectal cancer (CRC) is among the most prevalent and lethal malignancies worldwide, with nearly two million new cases and around one million deaths each year (Morgan et al., 2023). Despite progress in screening and therapeutic strategies, CRC remains a major clinical challenge. A significant fraction of patients are diagnosed at advanced stages–approximately 20% present with metastatic disease–for which 5-year survival plummets to near 10%–15% (Siegel et al., 2025). Standard treatments for metastatic CRC, including combinatorial chemotherapy and targeted agents (e.g., anti-EGFR and anti-VEGF therapies), have extended survival modestly, but durable remissions are rare (DeStefanis et al., 2019). Immunotherapy with immune checkpoint inhibitors has shown dramatic success in microsatellite instability-high subsets of CRC, yet the majority of microsatellite stable tumors respond poorly, underscoring the need for novel therapeutic approaches (Kong et al., 2024). In particular, new strategies are required to overcome drug resistance, metastatic progression, and tumor immune evasion that currently limit the efficacy of existing CRC treatments.

Against this backdrop, protein tyrosine phosphatases that aberrantly regulate oncogenic signaling and immune responses have emerged as compelling molecular targets in CRC (Tiganis and Tonks, 2026). PTPN1 (encoding protein tyrosine phosphatase 1B, PTP1B) and PTPN2 (encoding T-cell protein tyrosine phosphatase, TC-PTP) are two closely related non-receptor PTPs now recognized to play pivotal roles in cancer biology (Delibegović et al., 2024). These phosphatases normally function as negative regulators of tyrosine kinase signaling, maintaining cellular homeostasis by dephosphorylating key signaling proteins (Callahan et al., 2025). In CRC, however, their activity appears to be co-opted to drive malignancy. PTPN1 in particular is frequently overexpressed or amplified in colorectal tumors, correlating with more invasive disease and poorer patient survival (Li et al., 2025). Functionally, PTP1B has been implicated in sustaining pro-tumorigenic pathways–for example, by modulating growth factor receptor and oncogenic kinase signaling–thereby promoting cancer cell proliferation, migration, and metastasis (Delibegović et al., 2024). Likewise, mounting evidence indicates that PTPN2 can foster colorectal tumor development. Although historically considered a potential tumor suppressor due to its ability to restrain growth factor and cytokine signaling, PTPN2 has demonstrated context-dependent oncogenic effects (Zhang et al., 2025). High PTPN2 expression or activity in tumors has been associated with enhanced tumor progression, which is now understood to be linked to its profound impact on the tumor–immune interface.

Beyond their tumor-intrinsic signaling effects, PTPN1 and PTPN2 are crucial regulators of anti-tumor immunity (Baumgartner et al., 2023). These enzymes act as intracellular “checkpoints” that attenuate immune signaling pathways; PTPN1 and PTPN2 dephosphorylate components of the JAK–STAT cascade and T-cell receptor (TCR) signaling, thereby dampening interferon- and interleukin-mediated responses and limiting T cell activation (Wang et al., 2024). By shutting down cellular sensitivity to inflammatory cytokines, PTPN1/2 create an immunosuppressive milieu that allows cancer cells to escape immune surveillance (Tufail et al., 2025). Notably, loss-of-function studies have highlighted their role in immune evasion: genetic ablation of PTPN2 in tumor models leads to heightened immunogenicity and improved immune-mediated tumor clearance, and similar ablation in T cells unleashes more potent cytotoxic activity (Ott et al., 2023). In fact, genome-wide CRISPR screens first identified PTPN2 as a top target whose inhibition sensitizes tumors to immunotherapy, sparking interest in phosphatases as therapeutic targets (Manguso et al., 2017). Emerging preclinical evidence has now validated that inhibiting PTPN1 and PTPN2 can provoke a powerful dual anti-cancer response–simultaneously boosting the activity of effector immune cells (such as cytotoxic T lymphocytes and NK cells) and increasing tumor cell susceptibility to immune attack (Liang et al., 2023). In essence, blocking these two phosphatases releases brakes on the immune system while also preventing tumor cells from self-protecting against immune signals. Given that PTPN1 and PTPN2 share high homology and overlapping substrates, they can compensate for each other’s function to some extent; thus, dual inhibition is a rational strategy to achieve a more robust and durable therapeutic effect than targeting either enzyme alone. This dual-target approach promises to strike both pillars of CRC malignancy–the tumor’s intrinsic survival pathways and its extrinsic immune evasion mechanisms.

However, identifying potent small-molecule inhibitors against challenging enzyme targets like PTPN1 and PTPN2 demands innovative approaches. Protein tyrosine phosphatases have long been considered difficult to drug due to their highly conserved, positively charged active sites, which favor binding of phosphate-containing substrates and present selectivity and cell-permeability hurdles for drug development (Tiganis and Tonks, 2026). To surmount these challenges, we harnessed a comprehensive computer-aided drug design (CADD) strategy, leveraging state-of-the-art *in silico* techniques to discover and optimize potential dual inhibitors efficiently (Xu et al., 2024). CADD approaches have become indispensable in modern drug discovery, as they can rapidly explore vast chemical space and elucidate protein–ligand interactions with atomic detail.

**FIGURE 1:**
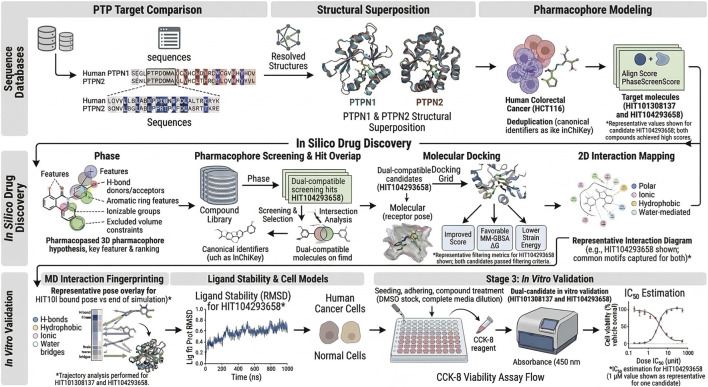
Research flow chart.

In this study, we integrated a multifaceted CADD workflow to identify and refine dual-target inhibitor candidates for PTPN1 and PTPN2: molecular docking was used to predict binding poses and rank affinities within both catalytic pockets, pharmacophore modeling was constructed from key interaction motifs (including donor/acceptor, aromatic, and ionizable features with steric constraints) to encode dual-recognition requirements, large-scale virtual screening was then performed to rapidly down-select high-confidence hits from vast chemical space, and explicit-solvent MD simulations were applied to top complexes to evaluate pose stability, capture induced-fit relaxation, and filter false-positive docked poses under near-physiological dynamics. Together, this docking–pharmacophore–screening–MD cascade creates a mutually reinforcing framework that increases confidence in prioritized hits before experimental testing and accelerates lead discovery by concentrating resources on the most dual-compatible chemotypes. Accordingly, applying this advanced *in silico* pipeline to CRC offers a timely strategy to explore drug-like dual inhibitors that could simultaneously blunt tumor cell signaling while alleviating immunosuppressive constraints in the tumor microenvironment, expanding the tractability of targets often viewed as challenging and supporting the development of multi-targeted, immune-enhancing therapeutic candidates. The workflow of our study is illustrated in Figure 1.

## 2 Materials and methods

### 2.1 Sequence alignment and domain comparison

Human PTPN1 and PTPN2 protein sequences were obtained from UniProt (https://www.uniprot.org/). Full-length global pairwise alignment was performed to evaluate overall similarity. To focus on the catalytic core, PTP domains were extracted according to annotated boundaries and aligned separately. Sequence identity and conservative-substitution similarity were computed over non-gap positions, with conservative substitutions defined using the BLOSUM62 matrix (Eddy, 2004).

### 2.2 Structural preparation and pocket comparison

Co-crystal structures of PTPN1 and PTPN2 catalytic domains were downloaded from the Protein Data Bank (PDB IDs: 8SKL and 9C55) (Bester et al., 2024; Liang et al., 2023). All structure processing was carried out in Schrödinger Suite 2025–3 (Maestro). Protein structures were prepared using the Protein Preparation Wizard, including assignment of bond orders, addition of hydrogen atoms, optimization of the hydrogen-bonding network, and restrained energy minimization. The OPLS4 force field (Memar and Moosavi, 2023) was used where applicable in the preparation and energy minimization steps. Catalytic domains were structurally aligned using Maestro Structural Alignment/Superposition based on backbone atoms to compare active-site geometry. Co-crystallized ligands were retained to enable direct binding-site and pose comparison.

### 2.3 Structure-based pharmacophore modeling

Structure-based 3D pharmacophore hypotheses were generated from the prepared co-crystal complexes using Phase (Schrödinger 2025–3). Key pharmacophore features (H-bond donors/acceptors, aromatic ring features, and ionizable groups) were automatically derived from observed ligand–protein interactions, and excluded-volume constraints were included to represent steric boundaries of the pocket (Vuorinen and Schuster, 2015). Hydrogen-bond features were treated as directional constraints, whereas aromatic/ionizable features were represented as plane/point features. Feature contributions were ranked according to the scoring output reported by Phase during hypothesis generation (Qingxin et al., 2025).

### 2.4 Pharmacophore screening and hit overlap

Pharmacophore-based screening was performed using Phase Ligand Screening in Schrödinger 2025–3. Hit lists were ranked by Align Score and PhaseScreenScore (Lanka et al., 2023). Prior to overlap analysis, screening outputs were standardized and deduplicated by chemical identity (e.g., canonical identifiers such as InChIKey), and the intersection of deduplicated hit sets was computed to obtain candidate dual-compatible molecules.

### 2.5 Docking and tri-metric post-docking filtering

Ligands were prepared using LigPrep (Schrödinger 2025–3), with protonation/tautomer states generated using Epik at a physiological pH range, followed by geometry optimization using OPLS4. Molecular docking was performed using Glide. Receptor docking grids were generated with Glide Receptor Grid Generation, centered on the co-crystallized ligand binding site for each target. Docking poses were further evaluated using Prime MM/GBSA to estimate binding free energy (ΔG), employing OPLS4 for energy evaluation. Ligand strain energy was computed within the Schrödinger workflow to quantify the conformational penalty associated with adopting the bound pose (Zhou et al., 2025). Compounds were prioritized using a tri-metric filter relative to the corresponding co-crystal inhibitor, requiring improved docking score, more favorable MM/GBSA ΔG, and lower strain energy.

### 2.6 Interaction mapping

Protein–ligand interaction analyses and 2D interaction diagrams were generated in Schrödinger 2025–3 using Maestro’s interaction visualization tools (e.g., Ligand Interaction Diagram). Interactions were summarized to capture polar/ionic/hydrophobic and water-mediated contacts, with emphasis on conserved PTP catalytic motifs (PTP loop/HCX5R, WPD loop, and Q-loop) (Crean et al., 2021; Pinkston et al., 2021).

### 2.7 Molecular dynamics simulations

MD simulations were performed using Desmond (Schrödinger 2025–3). Protein–ligand complexes were solvated using a standard explicit-solvent system builder, neutralized and ionized as needed, and equilibrated using the default Desmond relaxation protocol. Production simulations were conducted for 1 μs under standard ensemble control (e.g., NPT) using OPLS4-compatible parameters. For each protein-ligand complex, three independent MD replicates were performed with different random seeds (2007, 8871, and 14338). Trajectories were aligned to the protein backbone, and ligand pose stability was quantified by Lig fit Prot RMSD (ligand heavy-atom RMSD relative to the initial bound pose) using Schrödinger trajectory analysis tools.

### 2.8 MD interaction fingerprinting

Residue-wise interaction occupancy over MD trajectories was computed using Simulation Interactions Diagram (Schrödinger/Desmond trajectory analysis) and decomposed by interaction type, including hydrogen bonds, hydrophobic contacts, ionic interactions, and water bridges. Representative pose overlays were generated in Maestro by comparing the initial bound pose with a conformation extracted from the end of the simulation.

### 2.9 Cell culture and CCK-8 viability assay

Human colorectal cancer cell lines HCT116 and SW480 (Butler et al., 2017), and normal human colonic epithelial cell lines NCM460 and FHC (Moyer et al., 1996), were maintained under supplier-recommended culture conditions. In general, cells were cultured in the recommended complete media supplemented with fetal bovine serum (FBS) and antibiotics, and incubated at 37 °C in a humidified atmosphere with 5% CO_2_. Cells were routinely passaged at sub-confluent density and confirmed to be free of *mycoplasma* contamination prior to experiments.

Test compounds HIT101308137 and HIT104293658 were purchased from TargetMol. Stock solutions were prepared using 10% DMSO as the mother-solution vehicle and were diluted in complete medium immediately before use. Cells were treated with increasing concentrations of each compound for 48 h, and the same final vehicle proportion was maintained across all wells, including vehicle controls.

All conditions were tested with five independent replicates (n = 5), and results are presented as mean ± SD. Where applicable, dose–response curves were fitted using a nonlinear regression model (four-parameter logistic) to estimate IC_50_ values.

## 3 Results

### 3.1 Catalytic-domain conservation supports dual-target feasibility

**FIGURE 2:**
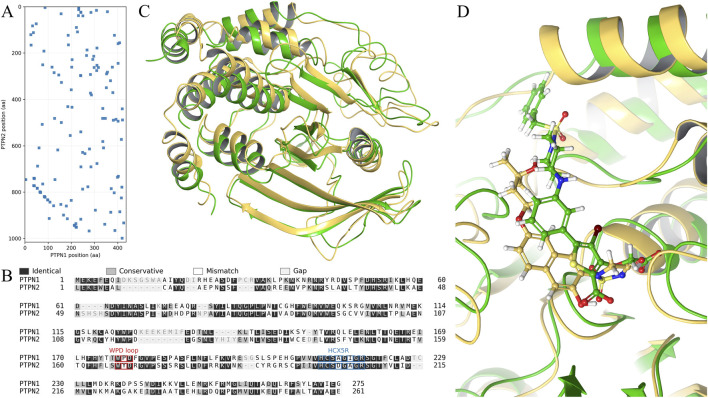
Stepwise sequence and structure comparison of PTPN1 and PTPN2 highlights catalytic-pocket conservation and supports the feasibility of dual targeting. **(A)** Full-length sequence comparison (dot-plot/alignment visualization) showing that similarity is concentrated in localized regions despite the pronounced length difference between PTPN1 (435 aa) and PTPN2 (1015 aa). **(B)** Pairwise alignment of the PTP (tyrosine-protein phosphatase) domain fragments, revealing conservation within the catalytic core. **(C)** Structural superposition of the catalytic PTP domains from PTPN1 (PDB ID: 8SKL) and PTPN2 (PDB ID: 9C55). PTPN1 is shown as a yellow cartoon, and PTPN2 is shown as a green cartoon. **(D)** Superposition of the ligand-binding regions with the co-crystallized inhibitors from 8SKL (PTPN1) and 9C55 (PTPN2). The PTPN1 co-crystal inhibitor is shown as a yellow stick model, and the PTPN2 co-crystal inhibitor is shown as a green stick model, indicating that the binding sites and ligand poses are largely overlapping.

To assess whether PTPN1 and PTPN2 could be jointly targeted by a dual inhibitor, we performed a stepwise comparison from full-length sequence alignment to PTP-domain alignment, followed by a structure-based evaluation of the catalytic pocket and ligand-binding region. The full-length proteins differ substantially in size (PTPN1: 435 aa; PTPN2: 1015 aa). As shown in Figure 2A, this pronounced length disparity suggests that, beyond the catalytic region, the two proteins likely contain distinct regulatory and/or structural modules. Global alignment of the full-length sequences showed that among the comparable non-gap positions (n = 274), the sequence identity was 44.53%, and the conservative-substitution similarity (BLOSUM62 score >0) was 60.95%. To evaluate conservation of the catalytic core, we further aligned the extracted PTP-domain fragments (PTPN1: 275 aa; PTPN2: 261 aa), as shown in Figure 2B. Among n = 200 comparable positions, the identity was 44.00% and the similarity was 59.20%.

We next compared the catalytic domains and compound-binding regions using available co-crystal structures of PTPN1 (PDB ID: 8SKL) and PTPN2 (PDB ID: 9C55) (Figures 2C,D). The overall fold and secondary-structure arrangement of the catalytic domains were highly consistent, and the ligand-binding regions were nearly superimposable, with the co-crystallized inhibitors adopting largely overlapping poses. mportantly, prior studies have reported high catalytic-domain similarity between these two phosphatases (e.g., ∼72–74% sequence identity), supporting the expectation that active-site–directed ligands may exhibit cross-reactivity. Moreover, key active-site motifs that define classical PTP catalysis—including the PTP signature loop (HCX5R/CX_5_R) and the mobile WPD loop—are strongly conserved in both structure and function. Collectively, although PTPN1 and PTPN2 diverge substantially at the full-length level, their catalytic pockets remain highly conserved, indicating that inhibitors targeting the catalytic pocket may display cross-binding/cross-inhibition—a challenge for selectivity optimization, but also a structural basis for dual-target coverage.

### 3.2 Structure-based pharmacophores define dual-compatible recognition patterns

To further formalize the interaction requirements within the catalytic pockets of PTPN1 and PTPN2, we generated structure-based 3D pharmacophore hypotheses from their respective co-crystal complexes. In this framework, pharmacophore features (e.g., hydrogen-bond donor/acceptor, aromatic ring, and ionizable groups) encode the key interaction pattern required for binding, while excluded-volume constraints represent steric boundaries of the pocket to improve screening specificity; hydrogen-bond features are commonly treated as directional (vector) constraints, whereas aromatic/ionizable features are represented as plane/point constraints.

**FIGURE 3:**
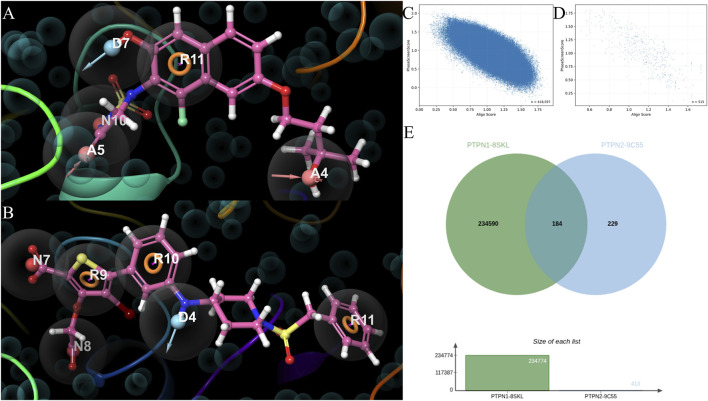
Structure-based pharmacophore hypotheses for PTPN1 and PTPN2 and pharmacophore-screening results. **(A)** Pharmacophore feature list/hypothesis derived for PTPN1. The model contains five features: R11 (aromatic ring), A4/A5 (H-bond acceptors), D7 (H-bond donor), and N10 (negative ionizable), with feature ranking indicating R11 as the dominant contributor. **(B)** Pharmacophore model derived for PTPN2. The model contains six features: R9/R10/R11 (aromatic rings), N7/N8 (negative ionizable), and D4 (H-bond donor), with aromatic ring features contributing most strongly. Excluded-volume spheres depict steric constraints imposed by the binding pocket. **(C)** Scatter plot of Align Score *versus* PhaseScreenScore from the PTPN1 pharmacophore screening output (compound identifiers not shown). **(D)** Scatter plot of Align Score *versus* PhaseScreenScore from the PTPN2 pharmacophore screening output (compound identifiers not shown). **(E)** Venn diagram summarizing the overlap of PTPN1 and PTPN2 pharmacophore hit lists.

For PTPN1, the hypothesis comprised five core features (Figure 3A): one aromatic ring feature (R11), two hydrogen-bond acceptors (A4 and A5), one hydrogen-bond donor (D7), and one negative-ionizable feature (N10). Feature ranking suggested that the aromatic anchor is the dominant contributor (R11, Score = −1.43), followed by A5 (−0.48), N10 (−0.27), A4 (−0.18), and D7 (−0.14), consistent with an active-site recognition pattern driven by aromatic/hydrophobic anchoring complemented by multi-point polar interactions.

For PTPN2, the hypothesis comprised six core features (Figure 3B), including three aromatic ring features (R9, R10, R11), two negative-ionizable features (N7 and N8), and one hydrogen-bond donor feature (D4). Aromatic features contributed most strongly (R9, −1.05; R10, −0.86; R11, −0.68), followed by N8 (−0.66), N7 (−0.53), and D4 (−0.32), indicating a binding pattern dominated by multi-ring anchoring together with phosphate-mimicking electrostatic requirements.

We next visualized the pharmacophore screening outputs in score space by plotting Align Score *versus* PhaseScreenScore for PTPN1 and PTPN2, respectively (Figures 3C,D; compound identifiers are omitted). Before overlap analysis, the hit lists were standardized and deduplicated by chemical identity (e.g., using standard InChI/InChIKey–based identifiers) to remove repeated entries that can arise from alternative representations of the same structure. Finally, the overlap of deduplicated hit sets is summarized in Figure 3E: the PTPN1 hypothesis retrieved 234,774 unique hits and the PTPN2 hypothesis retrieved 413 unique hits, with 184 shared molecules. Notably, the shared set accounts for 44.6% of the PTPN2 hit list (184/413), providing a focused pool of candidates compatible with dual PTPN1/PTPN2 active-site recognition, while differences in feature composition may offer handles for tuning selectivity *versus* dual-target coverage.

### 3.3 Tri-metric rescoring narrows dual-target candidates to three chemotypes

**FIGURE 4:**
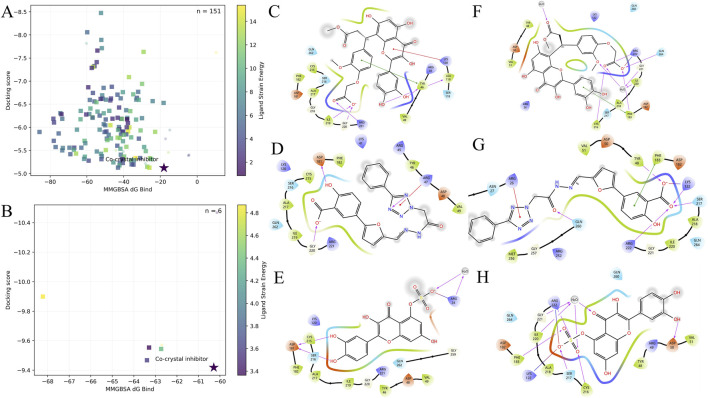
Tri-metric score landscapes and interaction analysis of shared candidates. **(A)** PTPN1: 2D landscape of all hits (n = 151) plotted as MM/GBSA ΔG_bind_ (x-axis) vs. docking score (y-axis; inverted for visualization), with ligand strain energy encoded by color. Squares indicate compounds that pass the tri-metric filter relative to the co-crystal reference inhibitor (more favorable ΔG_bind_ and docking score, and lower strain energy), whereas circles indicate compounds that fail the filter (≥1 metric not improved *versus* reference). The reference inhibitor is marked with a star. **(B)** PTPN2: same representation for all hits (n = 6); squares = tri-metric pass and circles = tri-metric fail relative to the reference; reference inhibitor marked with a star. **(C–E)** PTPN1 2D interaction maps for the three shared candidates (ID-based intersection), shown in order: **(C)** HIT101308137, **(D)** HIT104293658, **(E)** NP-023005. **(F–H)** PTPN2 2D interaction maps for the same three candidates, shown in order: **(F)** HIT101308137, **(G)** HIT104293658, **(H)** NP-023005. Interaction diagrams summarize polar/ionic and water-mediated contacts consistent with conserved PTP active-site motifs (PTP loop/HCX_5_R, WPD loop, and Q-loop).

Given the high conservation of the PTP catalytic pocket (PTP loop/HCX5R, WPD loop, and Q-loop) and our goal of prioritizing dual-target chemotypes, we conducted structure-based virtual screening against PTPN1 and PTPN2, followed by a tri-metric post-docking evaluation using docking score, Prime MM/GBSA ΔG_bind_, and ligand strain energy (Figure 4). Here, ΔG_bind_ is an end-point MM/GBSA estimate derived from the energetic difference between the complex and the separated receptor/ligand states, where more negative values generally indicate more favorable binding; ligand strain energy reflects the conformational penalty required for the ligand to adopt its bound-like geometry.

Figures 3A,B summarize all screened hits in a 2D score landscape (PTPN1: n = 151; PTPN2: n = 6), with ΔG_bind_ on the x-axis, docking score on the y-axis (inverted for visualization), and strain energy encoded by color. Using the co-crystal reference inhibitor as an internal benchmark, we applied a stringent tri-metric filter (ΔG_bind_ more favorable than reference, docking score more favorable than reference, and strain energy lower than reference). This excluded 11/151 PTPN1 hits (retaining 140) and 2/6 PTPN2 hits (retaining 4). The retained sets were then intersected by compound ID, yielding three shared candidates: HIT101308137, HIT104293658, and NP-023005.

To rationalize this ID-based overlap, we generated 2D interaction diagrams for the three candidates in both catalytic pockets (Figures 3C–H). Across both targets, the binding patterns align with canonical PTP active-site architecture: anionic/oxygen-rich groups engage the conserved HCX5R environment of the PTP loop, while additional polar and water-mediated contacts involve the WPD-loop Asp (general acid/base) and the Q-loop conserved Gln that helps position the catalytic water—together providing a structural basis for cross-target retention of these candidates.

### 3.4 MD reveals differential pose stability and interaction persistence in the PTPN1 pocket

**FIGURE 5:**
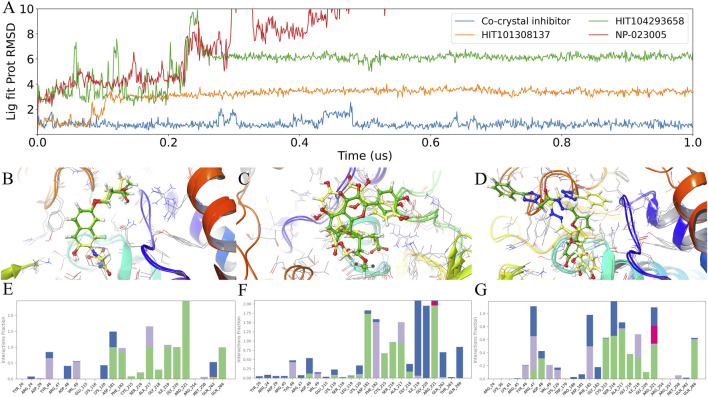
MD validation of ligand pose stability and interaction fingerprints in PTPN1. **(A)** Time evolution of Lig fit Prot RMSD for the co-crystal inhibitor and three candidate ligands over 1 μs MD simulations. RMSD was computed by aligning trajectory frames to the protein backbone and measuring ligand heavy-atom RMSD relative to the initial pose. **(B–D)** Representative binding pose overlays in the PTPN1 pocket comparing the pre-MD conformation (green) and the 1 μs conformation (yellow) for **(B)** co-crystal inhibitor, **(C)** HIT101308137, and **(D)** HIT104293658. **(E–G)** Residue-wise interaction occupancy (interaction fraction) during the MD simulations for **(E)** co-crystal inhibitor, **(F)** HIT101308137, and **(G)** HIT104293658. Stacked colors indicate interaction types: H-bonds (green), hydrophobic (purple), ionic (magenta), and water bridges (blue).

To further evaluate whether the shared candidates maintain a stable binding pose in the PTPN1 catalytic pocket, we carried out 1 μs MD simulations for the co-crystal reference inhibitor and the three candidates (HIT101308137, HIT104293658, and NP-023005). Ligand pose stability was quantified using Lig fit Prot RMSD, computed by aligning each trajectory frame to the protein backbone and measuring the RMSD of ligand heavy atoms relative to the initial bound conformation (Figure 5A). Overall, the co-crystal inhibitor was the most stable, maintaining consistently low RMSD (≈1 Å) with only brief transient excursions followed by rapid recovery. HIT101308137 showed an early adjustment phase and then converged to a moderate, relatively narrow plateau (≈3–4 Å), consistent with pose refinement within the pocket rather than progressive drift. In contrast, HIT104293658 exhibited a pronounced RMSD transition and subsequently stabilized at a higher plateau (≈6 Å), indicating a binding-mode switch followed by persistence in an alternative pose. NP-023005 displayed a sustained increase into a high-RMSD regime (≈8–10 Å) without convergence to a low/moderate plateau, suggesting weaker retention of the initial binding geometry.

These trends were supported by representative pose overlays comparing the pre-MD conformation (green) to the 1 μs conformation (yellow) (Figures 5B–D). The co-crystal inhibitor preserved a highly overlapping scaffold with only minor relaxation of peripheral substituents (Figure 5B). HIT101308137 remained within the same binding region but underwent a noticeable re-orientation consistent with its RMSD plateau behavior (Figure 5C). HIT104293658 showed the most substantial rearrangement, in line with the RMSD jump and higher plateau (Figure 5D).

To connect pose stability with engagement of functionally important residues, we computed residue-wise interaction occupancies across the trajectories and decomposed them by interaction type (Figures 4E–G; H-bonds, hydrophobic contacts, ionic interactions, water bridges, and halogen bonds). The co-crystal inhibitor exhibited the canonical interaction pattern, with high occupancies at the WPD-loop Asp181 and the PTP loop (Cys215–Arg221), dominated by hydrogen bonds and water bridges and featuring particularly strong engagement of Arg221 and neighboring P-loop residues (Figure 5E). HIT101308137 most closely recapitulated this reference network, showing robust P-loop anchoring (notably Gly220/Arg221) with persistent hydrogen-bond/water-bridge contributions while retaining interactions involving Asp181, consistent with a stable inhibitory binding mode in the catalytic pocket (Figure 5F). By comparison, HIT104293658 maintained P-loop contacts but shifted interaction emphasis toward the pTyr recognition/entrance region (Tyr46/Arg47/Asp48), with reduced sustained engagement of Asp181—consistent with its higher-RMSD plateau and the pose rearrangement observed in the overlays (Figure 5G). Given the pronounced pose drift of NP-023005, interaction-fingerprint analysis was therefore focused on the two candidates showing converged binding modes (HIT101308137 and HIT104293658) alongside the co-crystal reference.

### 3.5 PTPN2 MD fingerprints differentiate core-coupled vs. entrance-biased binding

**FIGURE 6:**
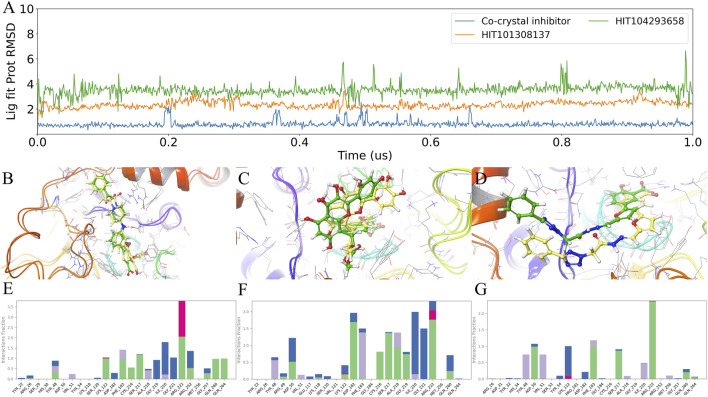
MD-based binding stability and interaction fingerprints in the PTPN2 catalytic pocket. **(A)** Time evolution of ligand pose stability during 1 μs MD simulations (Lig fit Prot RMSD), where trajectories are aligned to the protein backbone and ligand heavy-atom RMSD is computed relative to the initial bound pose. **(B–D)** Representative binding pose overlays comparing the pre-MD conformation (green) and the 1 μs conformation (yellow) for the ligands analyzed in PTPN2. **(E–G)** Residue-wise interaction occupancy (interaction fraction) over the 1 μs trajectories for **(E)** the PTPN2 co-crystal inhibitor, **(F)** HIT101308137, and **(G)** HIT104293658. Stacked colors denote interaction types: H-bonds (green), hydrophobic (purple), ionic (magenta), and water bridges (blue).

To characterize how the shared candidates engage the PTPN2 catalytic pocket under dynamic conditions—and whether they preserve a co-crystal–like interaction pattern relevant for dual-target design—we analyzed 1 μs MD trajectories using (i) ligand pose stability metrics together with representative pose overlays (Figures 6A–D), and (ii) residue-wise interaction occupancy decomposed by interaction type (Figures 6E–G). Here, *interaction fraction* denotes the fraction of simulation frames in which a given residue forms a specific contact with the ligand, and stacked bars indicate that multiple interaction types can be sampled by the same residue over the trajectory.

As shown in Figures 6A–D, the MD time series and pose overlays provide a qualitative view of binding-mode retention and conformational relaxation within the active-site region, which motivates the subsequent interaction-fingerprint comparison against the co-crystal reference. In addition, for PTPN1-HIT101308137, PTPN1-HIT104293658, PTPN2-HIT101308137, and PTPN2-HIT104293658, two independent MD replicates were performed under identical simulation conditions using different random seeds (8871 and 14338), together with the original run (seed 2007), yielding three RMSD time-evolution profiles per complex (Supplementary Figures S2–S5). Across replicates, both compounds remained highly stable in PTPN1, whereas PTPN2 showed moderate dynamic fluctuations; however, the fluctuation range was small and remained within an acceptable level. The co-crystal inhibitor interaction fingerprint (Figure 6E) displays a canonical PTP binding pattern centered on the PTP loop/P-loop and the conserved basic anchor Arg222. Arg222 shows the strongest overall occupancy, combining prominent ionic and H-bond contributions, consistent with stable recognition of an anionic/oxygen-rich ligand moiety at the catalytic pocket base. Surrounding P-loop residues (Cys216–Ser217–Ala218–Gly219–Ile220–Gly221) contribute additional H-bonds and water bridges, indicating a persistent polar/water-mediated network that helps lock the ligand in the catalytic cleft. The WPD-loop Asp182 also contributes detectable polar/water-mediated contacts, consistent with engagement of catalytically relevant elements beyond simple steric occupancy.

HIT101308137 (Figure 6F) most closely recapitulates this co-crystal–like network. It retains strong anchoring at Arg222 (again supported by H-bonds/ionic components) and reinforces the pocket-base network through high occupancies at Ile220/Gly221, particularly via water bridges. Importantly, HIT101308137 maintains clear contacts involving Asp182, supporting a binding mode that remains coupled to the WPD region rather than shifting primarily toward the entrance. Additional occupancy around Ser217/Ala218/Gly219 further supports stable positioning along the P-loop contour.

By contrast, HIT104293658 (Figure 6G) preserves the key Arg222 anchor—indicating that it can occupy the catalytic region—but shows a more entrance-influenced interaction distribution. Relative to HIT101308137, it exhibits stronger and more frequent contributions from residues in the entrance/pTyr-recognition region (e.g., Tyr48, Asp50, Val51), suggesting a binding mode that relies more on entrance-side contacts and is therefore more compatible with pose reorganization. While P-loop residues remain involved, the relative contribution from Asp182 is less pronounced, consistent with weaker or less persistent coupling to this catalytic element.

### 3.6 Early drug-likeness and medicinal-chemistry liability assessment

To provide translational context for the prioritized dual-target hits, we performed an *in silico* drug-likeness and medicinal-chemistry liability assessment. Both compounds were predicted to be synthetically accessible (SAscore: Easy, GASA: Easy), but they showed distinct developability profiles.

For HIT101308137, the profile indicates high polarity and limited oral-like space (MW = 568.12, TPSA = 213.42 Å^2, nHD = 6), with Lipinski Rule: Rejected and a low QED = 0.127. Although lipophilicity was moderate (logP = 1.793, logD7.4 = 1.868) and predicted solubility was acceptable (logS = −3.449), medicinal-chemistry alerts were present (PAINS = 1, Alarm_NMR = 3, BMS = 1, Chelating Rule = 2), suggesting non-negligible structural liability risk.

For HIT104293658, physicochemical properties were comparatively more favorable (MW = 432.12, nHD = 3, Lipinski Rule: Accepted, GoldenTriangle: Accepted, QED = 0.296), despite relatively high polarity (TPSA = 155.73 Å^2) and lower predicted solubility (logS = −4.374). This compound showed fewer structural alerts (PAINS = 0, BMS = 0), but high interference-risk scores (Colloidal aggregators = 0.999, FLuc inhibitors = 0.992, Blue fluorescence = 0.88, Green fluorescence = 0.975), indicating that orthogonal assay validation is necessary to exclude assay artifacts.

### 3.7 CCK-8 profiling suggests a preliminary tumor–normal selectivity window

**FIGURE 7:**
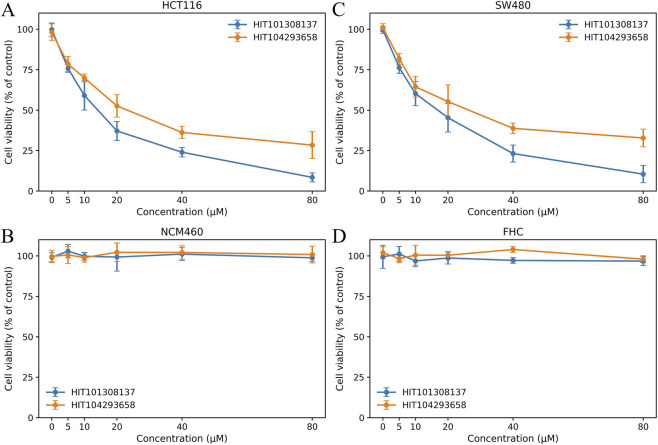
Dose–response effects of HIT101308137 and HIT104293658 on viability of colorectal cancer and normal colorectal cells. CCK-8 assays were performed in human colorectal cancer cell lines HCT116 **(A)** and SW480 **(B)**, and normal human colorectal epithelial cell lines NCM460 **(C)** and FHC **(D)**. Cells were treated with increasing concentrations of HIT101308137 or HIT104293658, and viability was normalized to vehicle-treated controls. Data are presented as mean ± SD (n = 5).

To preliminarily validate the anti-tumor activity of the two prioritized hits at the cellular level—and to assess their selectivity toward malignant *versus* non-malignant colonic cells—we quantified dose–response effects on cell viability using CCK-8 assays in two colorectal cancer (CRC) cell lines (HCT116 and SW480) and two normal colonic epithelial cell lines (NCM460 and FHC (Figure 7). The CCK-8 assay provides a colorimetric readout based on WST-8 reduction and is generally proportional to the number of metabolically active cells.

As shown in Figures 7A,B, both HIT101308137 and HIT104293658 induced a clear, concentration-dependent reduction in cell viability in HCT116 and SW480 cells, demonstrating reproducible *in vitro* anti-proliferative activity across CRC models. Importantly, the two compounds exhibited distinct potency profiles in tumor cells. HIT101308137 consistently produced stronger growth inhibition at matched concentrations, with a steeper decline in viability, whereas HIT104293658 displayed a more moderate but still monotonic dose–response behavior. Quantitative analysis indicates approximate IC_50_ values of ∼25 µM for HIT101308137 and ∼30 µM for HIT104293658 in both CRC cell lines, underscoring the superior cellular potency of HIT101308137 while confirming measurable activity for both hits.

In contrast, in normal colonic epithelial cells (NCM460 and FHC; Figures 7C,D), cell viability remained broadly close to vehicle-control levels across the tested concentration range for both compounds. Although increased within-group variability was observed in the updated normal-cell datasets, these fluctuations were centered around baseline viability rather than following a concentration-dependent decline, suggesting that neither compound induces marked cytotoxicity in non-malignant colonic cells under the same assay conditions.

Taken together, these results demonstrate that both prioritized hits suppress CRC cell viability while exerting minimal effects on normal colonic epithelial cells, supporting the presence of a preliminary selectivity window. At the same time, the overall magnitude of growth inhibition remains moderate, indicating that further optimization of chemical structure and physicochemical properties will likely be required to enhance cellular potency while preserving low toxicity toward normal cells.

## 4 Discussion

This study links cross-target pocket conservation to actionable ligand prioritization by integrating pharmacophore overlap, tri-metric post-docking filtering, microsecond-scale dynamics, and cellular viability profiling. Starting from a shared hit set, tri-metric rescoring reduced the search space to three dual-compatible candidates, and MD then resolved clear separation in binding-mode retention. Across both targets, stable occupation of the catalytic cleft was associated with persistence of a pocket-base anchor and sustained coupling to catalytic loops, whereas increased pose plasticity coincided with a redistribution of contacts toward the pocket entrance. In parallel, CCK-8 assays provided preliminary phenotypic support for a tumor–normal window, while also indicating that cellular potency remains modest and therefore likely optimization-limited rather than target-limited.

Our findings are consistent with—and extend—recent reports demonstrating that dual active-site inhibition of PTPN1/PTPN2 is pharmacologically tractable and can deliver anti-tumor activity *in vivo*. ABBV-CLS-484 (AC484) was reported as a first-in-class, orally bioavailable dual inhibitor that amplifies interferon responses and promotes immune-dependent tumor control, including models resistant to PD-1 blockade (Baumgartner et al., 2023). In a separate study, Compound-182 was described as a potent active-site dual inhibitor that enhances antigen-driven T-cell activation and suppresses tumor growth, including the MC38 colorectal model, without overt immune-related toxicities in the reported preclinical settings (Liang et al., 2023). Relative to these benchmarks, our work sits earlier in the discovery continuum: it emphasizes structural and dynamic determinants of dual compatibility and provides initial cellular phenotypes that can guide subsequent medicinal-chemistry and mechanism-of-action validation.

These drug-likeness results refine the interpretation of our cellular findings. Although both hits showed dual-target-compatible binding dynamics and preliminary anti-proliferative activity, their developability liabilities differ. HIT101308137 appears constrained mainly by high polarity and rule-of-five deviations, which may reduce intracellular exposure and contribute to modest cellular potency. HIT104293658 occupies a more drug-like physicochemical window, but its high predicted aggregation/fluorescence-interference risk necessitates careful orthogonal confirmation in non-fluorescence and anti-aggregation assay settings. Overall, both molecules are best positioned as lead-like starting points rather than development candidates at this stage, with optimization priorities focused on improving exposure-relevant properties while reducing assay-liability features.

To contextualize the relatively high concentrations used in our cell-viability assays, we benchmarked our hits against previously reported dual PTPN1/PTPN2 active-site inhibitors. Osunprotafib (ABBV-CLS-484), a clinically oriented oral dual inhibitor, has reported biochemical potency in the low-nanomolar range (PTPN1 IC_50_ = 2.5 nM; PTPN2 IC50 = 1.8 nM). Tegeprotafib (Compound 124) was reported with PTPN2 IC_50_ = 4.4 nM and PTPN1 IC50 in the 1–10 nM range, while TC-PTP-IN-1 (Compound 8) showed strong dual-target activity with a reported PTPN2 IC_50_ of 9.2 nM. Relative to these optimized benchmarks, our prioritized compounds should be considered lead-like starting points: they show dual-target-compatible binding dynamics and preliminary cellular activity, but still require further medicinal-chemistry optimization to improve biochemical potency and translate to stronger cellular efficacy.

The pharmacophore overlap analysis adds an important constraint that is often underappreciated in PTP-directed campaigns (Zhu et al., 2023). The markedly smaller PTPN2 hit set compared with PTPN1 indicates that PTPN2 imposes tighter feature compatibility under the same hypothesis-generation and screening logic, making the shared intersection a stringent subset rather than a trivial overlap. This feature-based narrowing is particularly valuable for catalytic PTP pockets, where purely score-driven workflows can enrich for highly polar ligands that exploit electrostatics yet are less likely to translate into robust, drug-like binding. The subsequent tri-metric gate further strengthened prioritization by requiring simultaneous improvement over a co-crystal benchmark in docking score, MM/GBSA ΔGbind, and conformational strain (Hou et al., 2011; Islam et al., 2025), thereby disfavoring candidates whose apparent affinity is driven by unstable poses or high deformation penalties.

Microsecond MD trajectories were decisive for distinguishing candidates with superficially similar static docking behavior. In both targets, the interaction fingerprints converged on an Arg-centered pocket-base anchor within the PTP loop, consistent with the catalytic signature’s role in stabilizing anionic/oxygen-rich moieties and the broader catalytic architecture involving the WPD loop and Q-loop. However, the candidates diverged in how this anchor was embedded within a stabilizing network over time. HIT101308137 maintained a more co-crystal-like, catalytic-core–coupled interaction topology, including persistent water-mediated stabilization along the P-loop contour and sustained engagement of the WPD-loop Asp. In contrast, HIT104293658 retained the pocket-base anchor but redistributed interaction occupancy toward the entrance/pTyr-recognition region, consistent with a binding-mode reorganization and higher RMSD plateaus. This “core-coupled *versus* entrance-biased” split provides a practical structural hypothesis for dual-target optimization: maximizing persistence of catalytic-core coupling may improve cross-target robustness, whereas entrance-side reliance may increase pose adaptability and therefore offer a potential handle for tuning selectivity if needed.

The cellular data support dual-target progression while clarifying the current ceiling. Both prioritized hits reduced CRC cell viability in a dose-dependent manner with limited effects in normal epithelial cells in the same assay window, suggesting a preliminary selectivity margin. At the same time, the moderate magnitude of growth suppression is compatible with a well-known challenge for active-site PTP ligands: achieving catalytic-site electrostatic complementarity often increases polarity and ionization, which can reduce intracellular exposure and dampen apparent cellular efficacy even when target-proximal binding is favorable. The MD-derived interaction maps suggest concrete design directions to address this gap: preserve the Arg-anchoring motif required for catalytic-site occupancy, reinforce WPD-region coupling and the pocket-base water network that correlates with pose retention, and pursue phosphate-mimicking strategies that reduce excessive anionic burden while maintaining geometry and hydrogen-bonding capacity.

A further contextual point is that dual inhibition is strongly linked to immune sensitivity in genetic and pharmacologic literature, which defines clear next-step assays beyond viability. *In vivo* CRISPR screening identified PTPN2 loss in tumor cells as a determinant of increased responsiveness to immunotherapy through enhanced interferon-γ–mediated antigen presentation and growth suppression. Together with the dual-inhibitor case studies above, this supports extending our validation to biochemical inhibition for both targets, broader phosphatase selectivity profiling, and cellular target-engagement readouts. It also motivates immune-relevant functional testing, including interferon-response amplification, T-cell activation phenotypes, and combination studies with checkpoint blockade, to determine whether the prioritized chemotypes reproduce the mechanistic hallmarks reported for clinically oriented dual inhibitors. An important limitation of the present work is that direct biochemical target validation for PTPN1/PTPN2 is not yet complete. We attempted SPR-based binding confirmation for both targets; however, despite extensive optimization, we were unable to establish sufficiently stable and reproducible sensor-chip immobilization conditions for reliable kinetic quantification. Therefore, the current dual-target conclusion is supported by convergent computational and cellular phenotypic evidence rather than definitive biophysical target-engagement data. In follow-up studies, we will prioritize orthogonal validation, including enzymatic inhibition assays for both phosphatases, selectivity profiling across related phosphatases, and cellular target-engagement assays, to further strengthen mechanistic confidence.

In summary, our data argue that dual-target feasibility is not solely a question of pocket similarity but of dynamic network persistence within the catalytic cleft. HIT101308137 emerges as the more catalytic-core–coupled scaffold across both targets, aligning with stronger pose retention and a co-crystal-like interaction footprint, whereas HIT104293658 samples a more entrance-biased mode that may be informative for tuning interaction distribution during SAR. Placed alongside AC484 and Compound-182, these results position our candidates as lead-like starting points and define concrete structural and experimental criteria for advancing dual-target inhibitors toward mechanism-linked efficacy.

## Data Availability

The raw data supporting the conclusions of this article will be made available by the authors, without undue reservation.
