# Supplementary figures and images for: HIT101308137 and HIT104293658 nominate dual target chemotypes for PTPN1 and PTPN2 with preliminary selectivity in colorectal cancer cells
Source: Front Chem. 2026 Apr 17;14:1782252. doi: 10.3389/fchem.2026.1782252 (PMC13133049; doi:10.3389/fchem.2026.1782252)

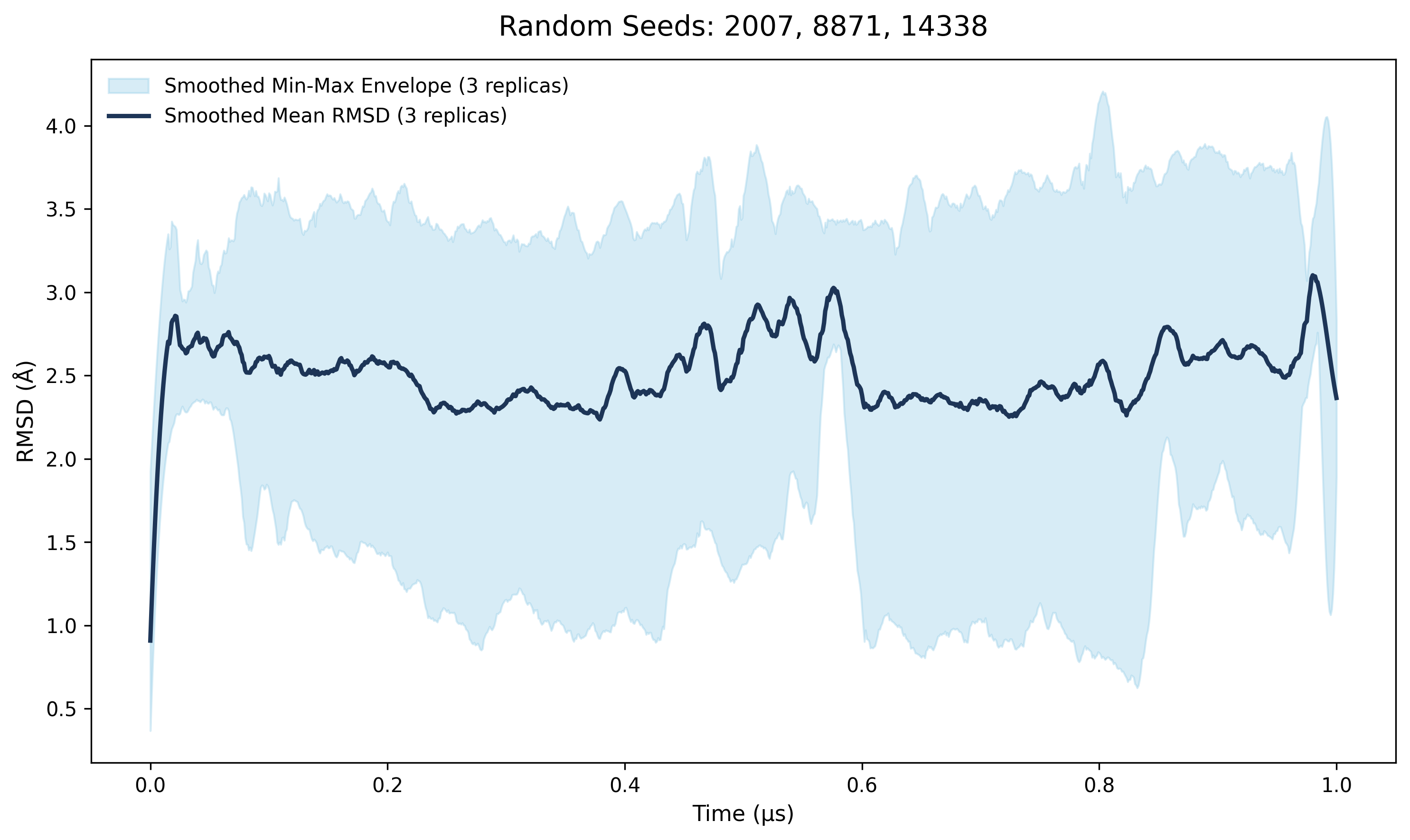

Supplement: Supplementary file 1 [file Image4.png]

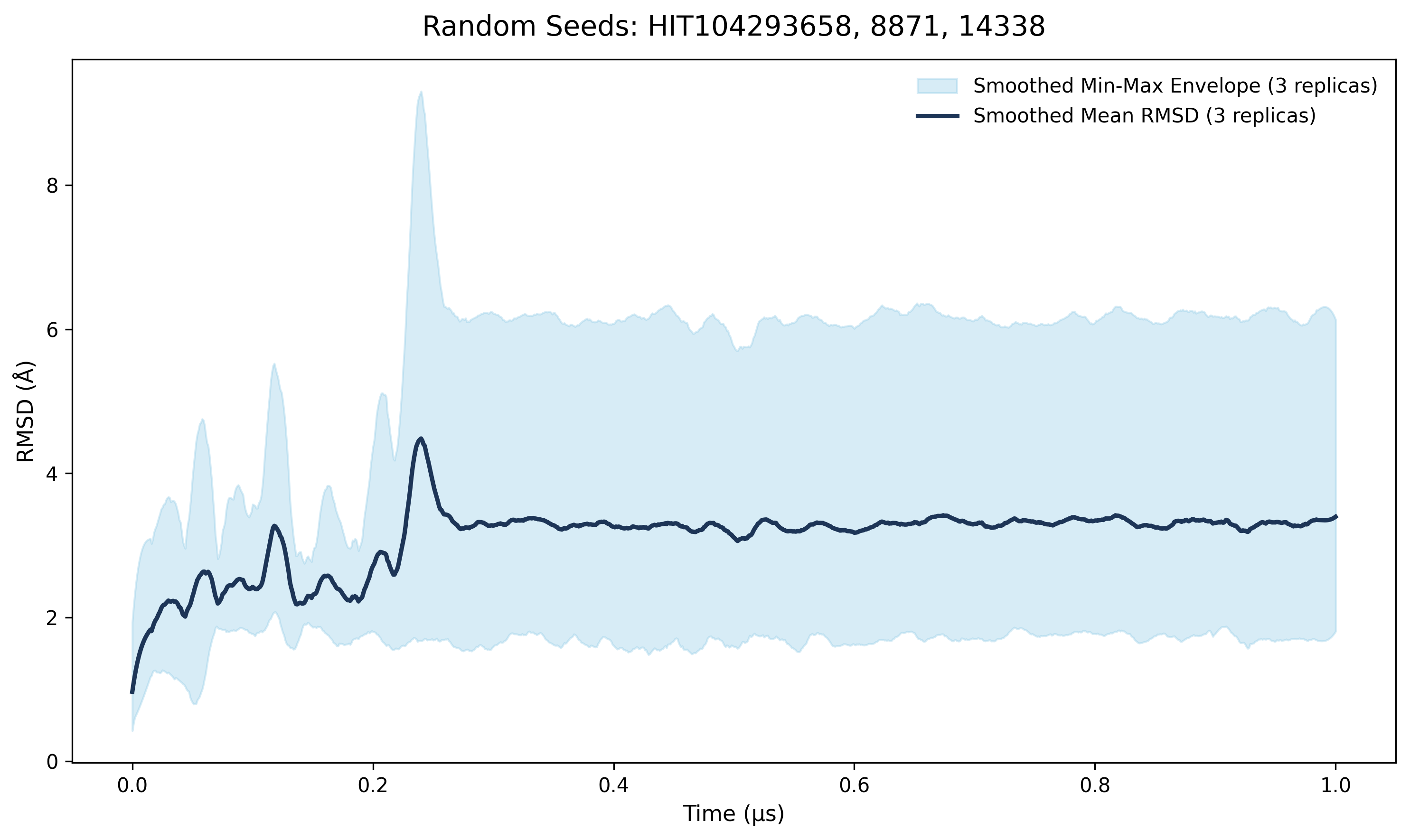

Supplement: Supplementary file 3 [file Image2.png]

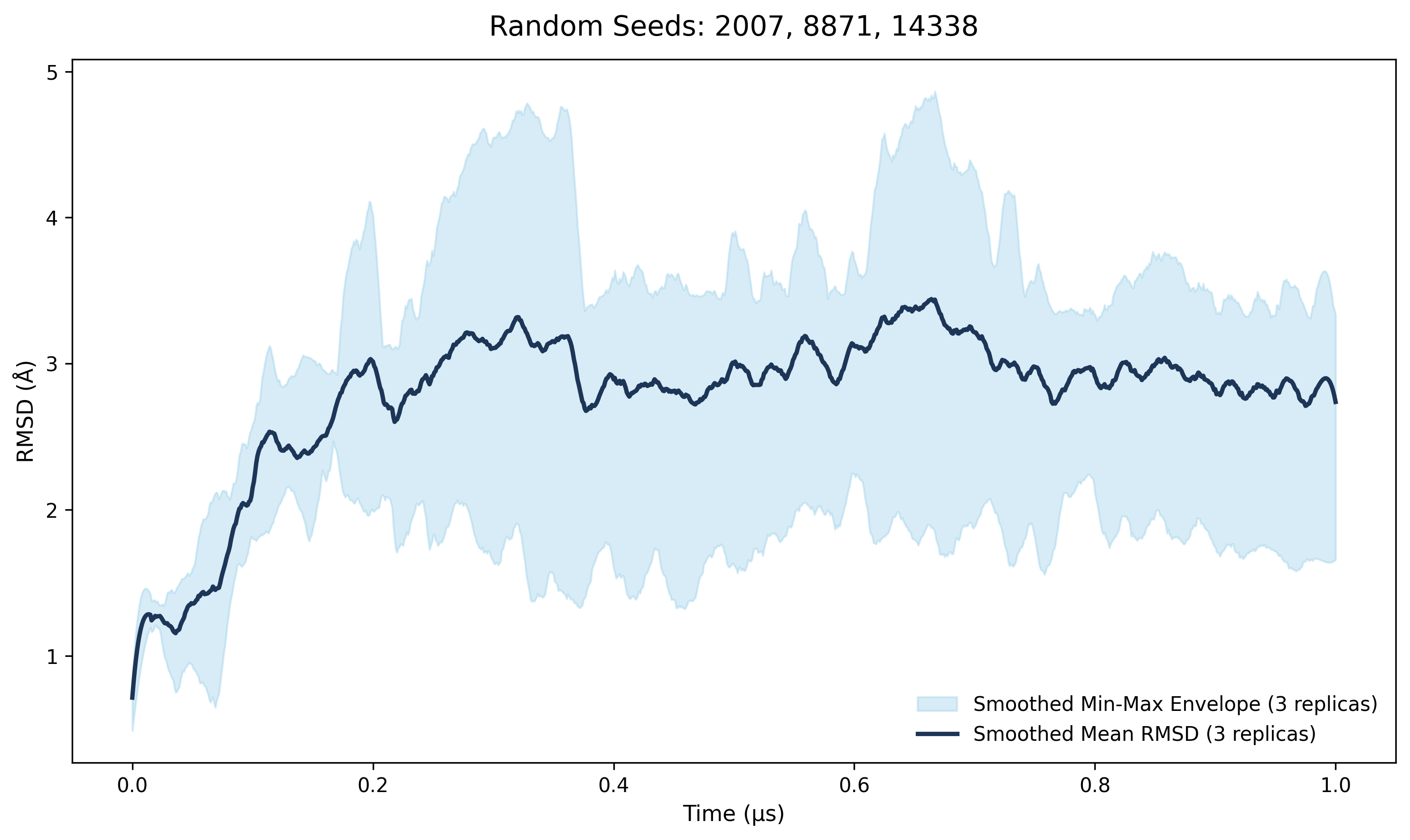

Supplement: Supplementary file 4 [file Image1.png]

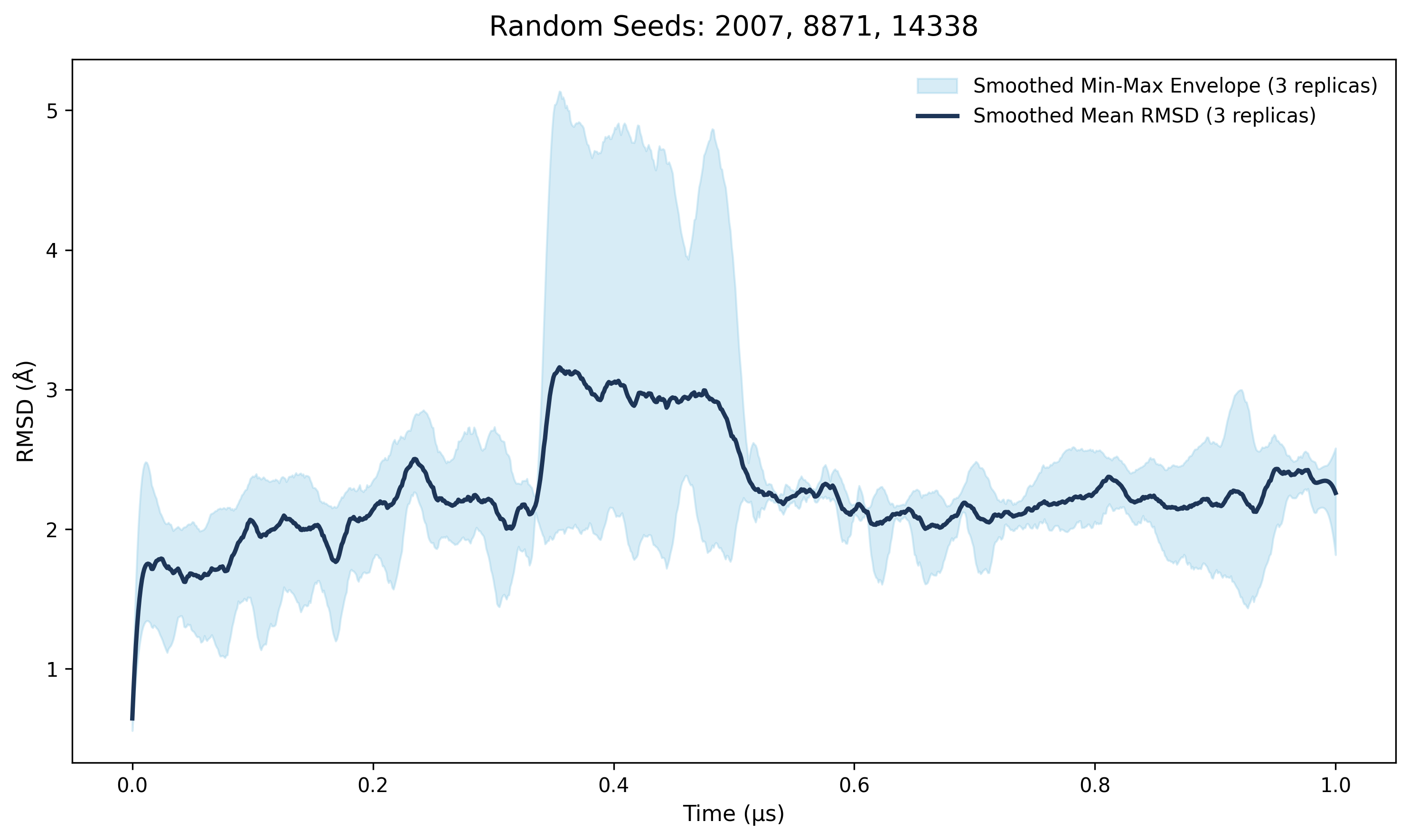

Supplement: Supplementary file 5 [file Image3.png]
