# Supplementary material for: HIT101308137 and HIT104293658 nominate dual target chemotypes for PTPN1 and PTPN2 with preliminary selectivity in colorectal cancer cells
Source: Front Chem. 2026 Apr 17;14:1782252. doi: 10.3389/fchem.2026.1782252 (PMC13133049; doi:10.3389/fchem.2026.1782252)
Supplement: Supplementary file 2 [file DataSheet1.pdf]

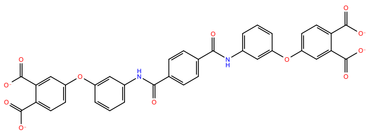

title HIT105782373

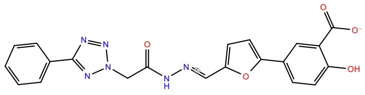

title HIT104293658

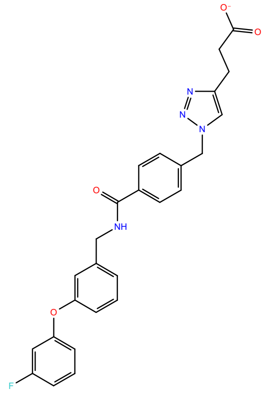

title HIT214735606

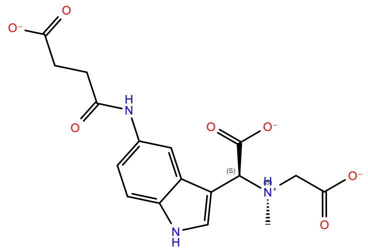

title STOCK6S-78805

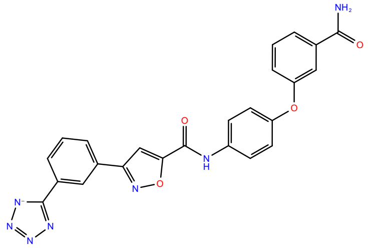

title HIT213743614

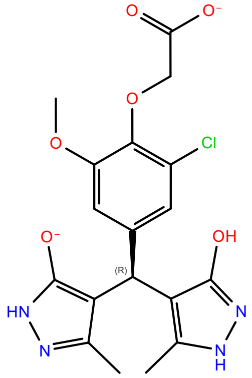

title HIT106686127

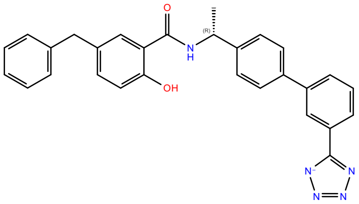

title HIT213730271

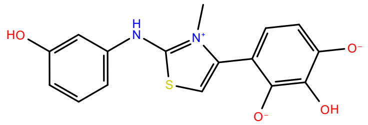

title STOCK7S-21022

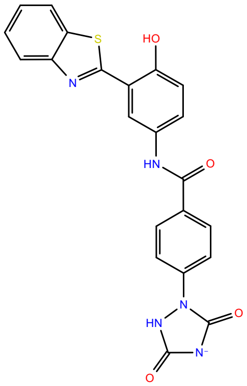

title HIT214567610

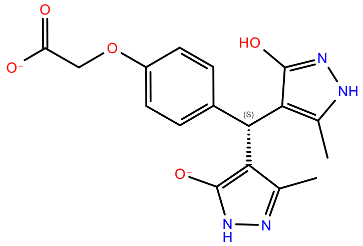

title HIT104457153

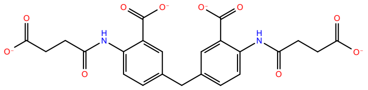

title HIT107099173

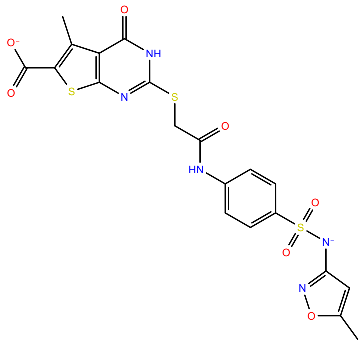

title F1722-0014

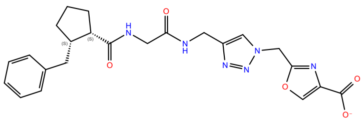

title HIT211671580

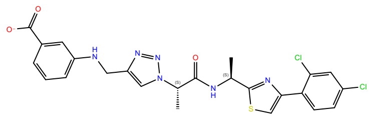

title HIT214669198

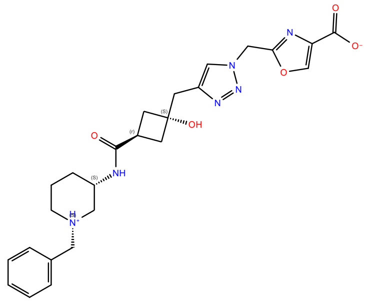

title HIT214735591

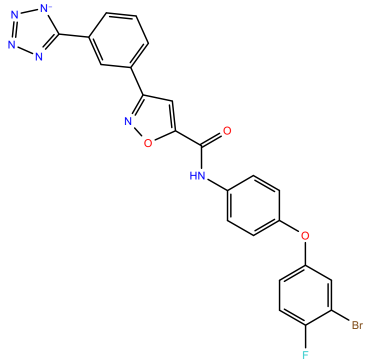

title HIT214690343

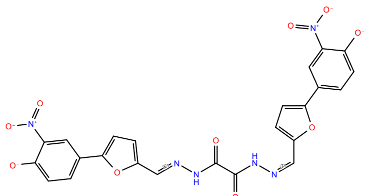

title HIT211749429

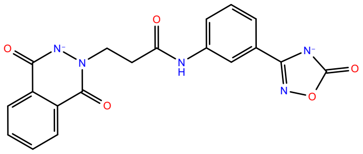

title HIT214741921

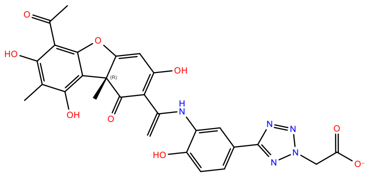

title HIT107398577

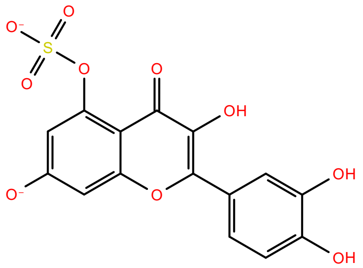

title NP-023005

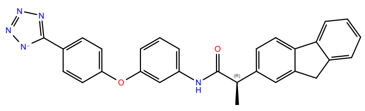

title HIT213730286

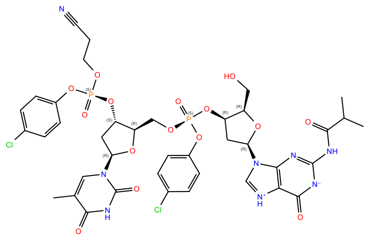

title HIT103692565

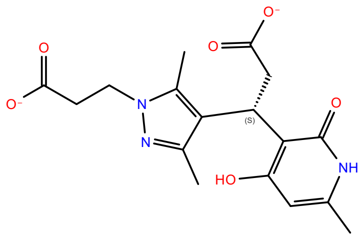

title HIT100232394

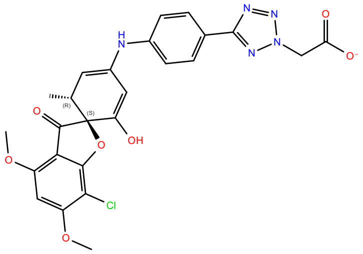

title HIT105089373

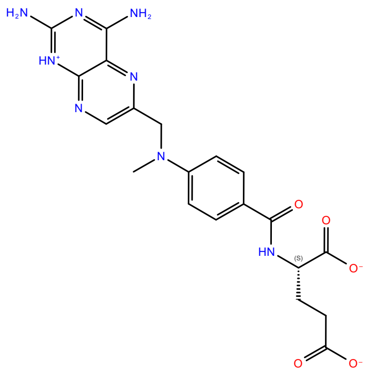

title HIT212083377

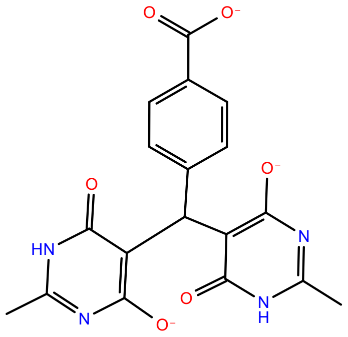

title HIT105298636

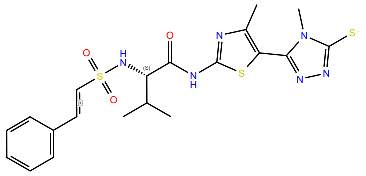

title HIT214811307

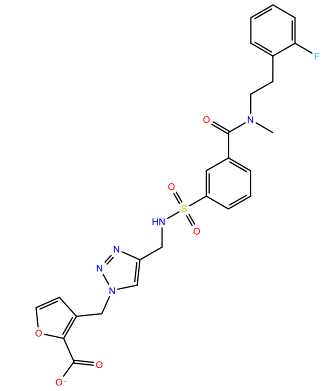

title HIT106763415

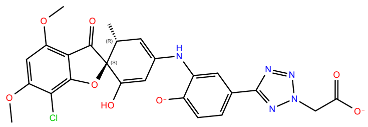

title HIT102538494

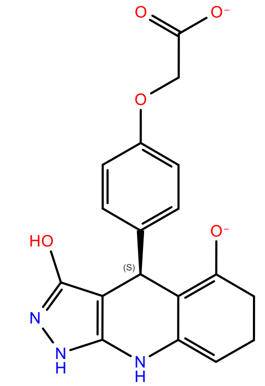

title HIT104285877

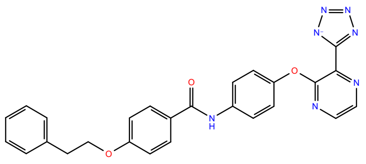

title HIT214660477

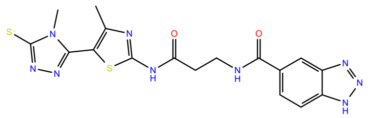

title HIT211568621

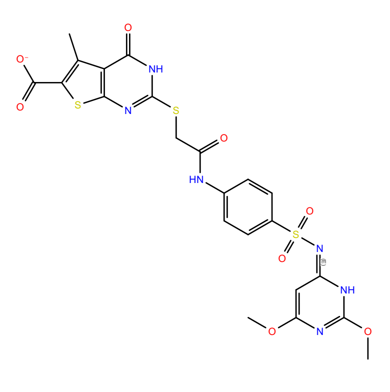

title F1634-0012

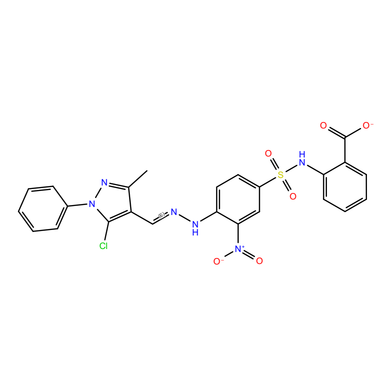

title HIT212597058

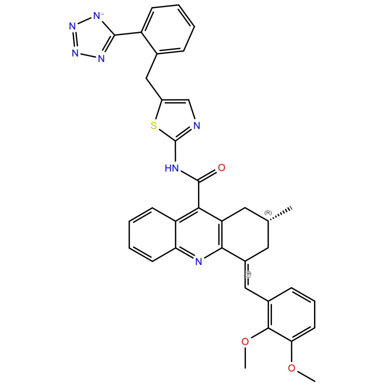

title HIT214677746

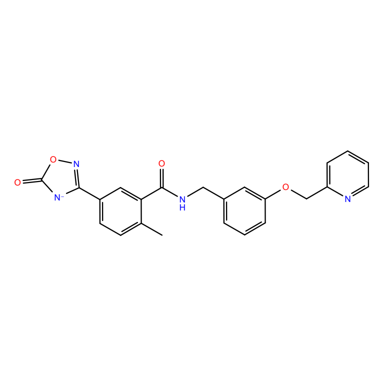

title HIT213728688

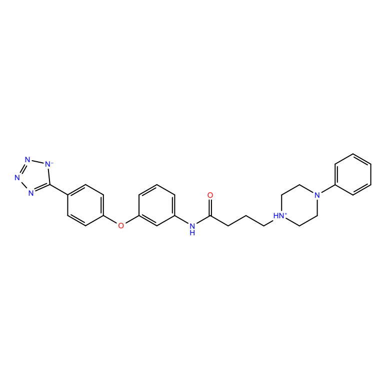

title HIT213714073

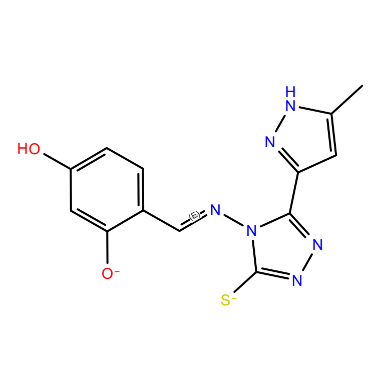

title HIT100778541

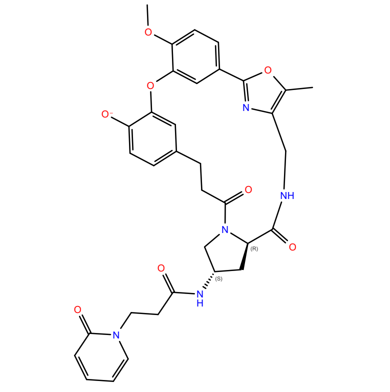

title HIT213843325

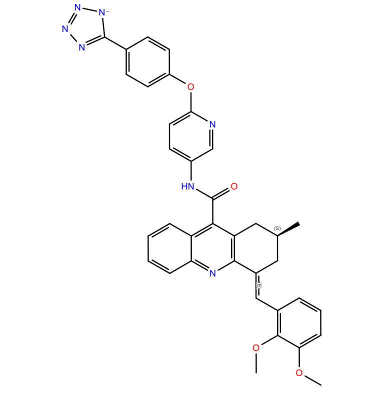

title HIT213725037

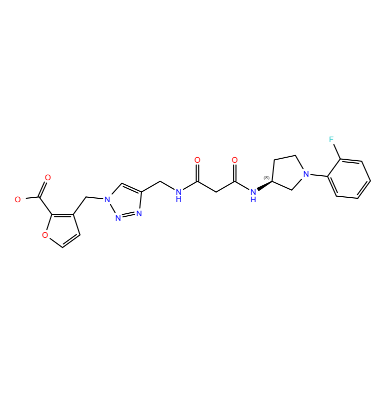

title HIT214665354

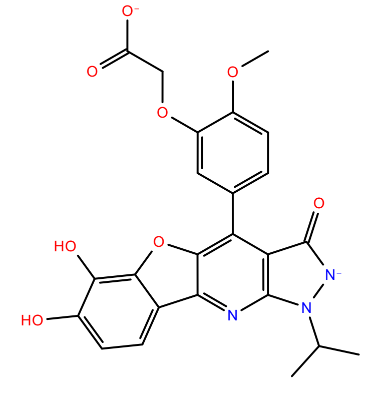

title HIT215011985

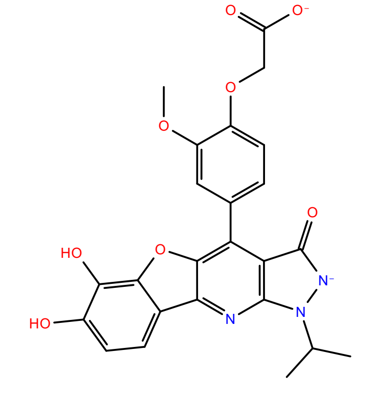

title HIT215012029

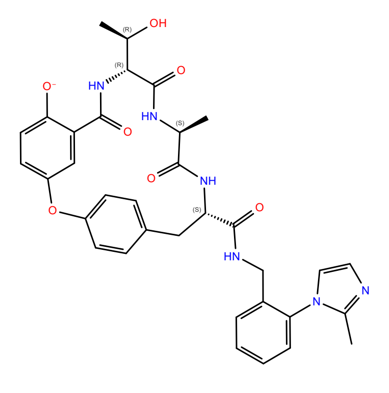

title HIT214980754

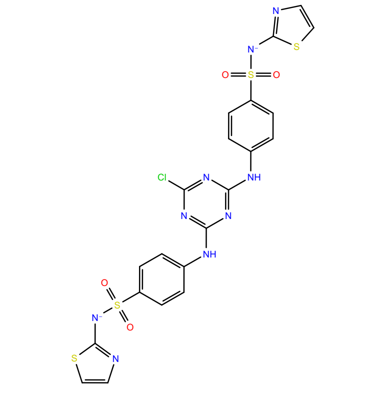

title STOCK7S-69637

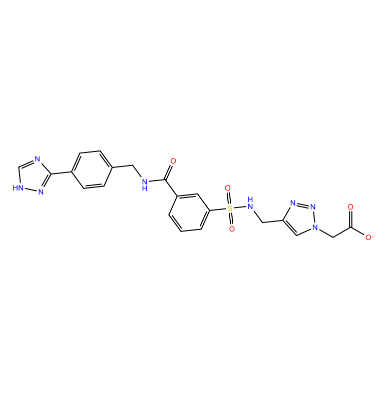

title HIT214665346

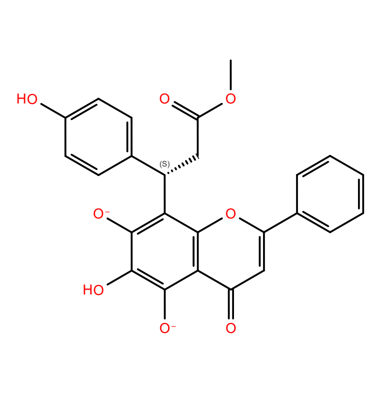

title HIT105089366

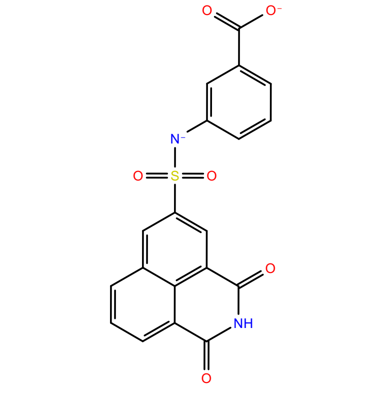

title HIT107185981

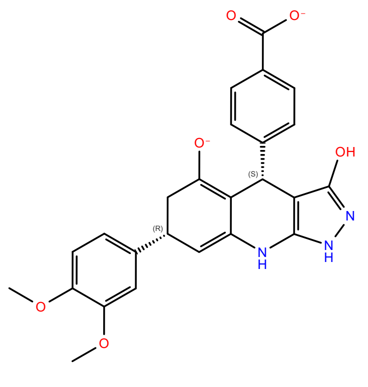

title HIT100001052

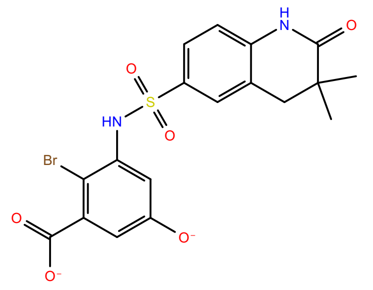

title HIT214678845

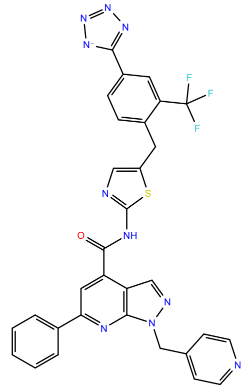

title HIT213717645

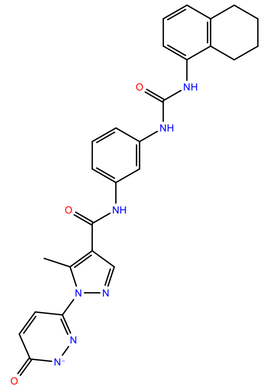

title HIT214804728

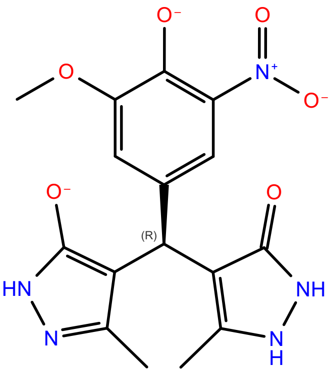

title HIT106592339

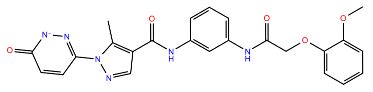

title HIT214757270

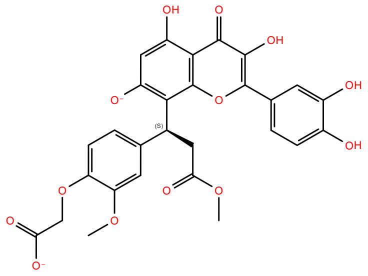

title HIT101308137

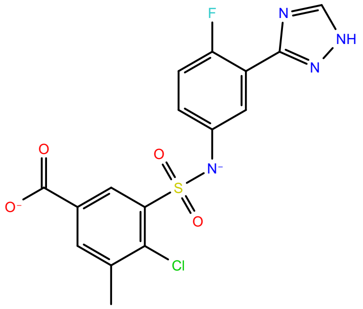

title HIT102238230

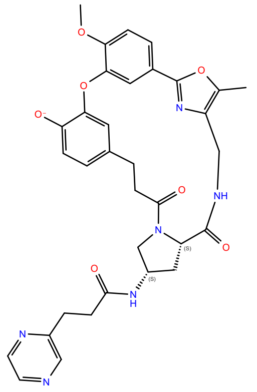

title HIT213842921

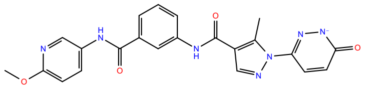

title HIT107431188

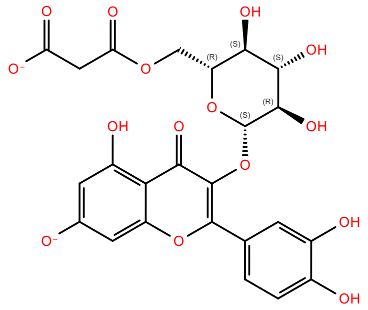

title NP-000435

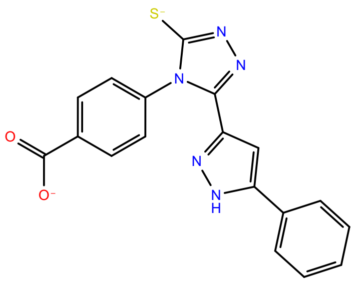

title HIT104270596

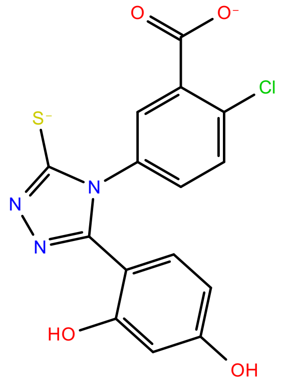

title HIT107129530

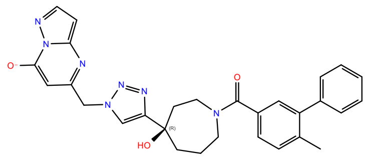

title HIT214679589

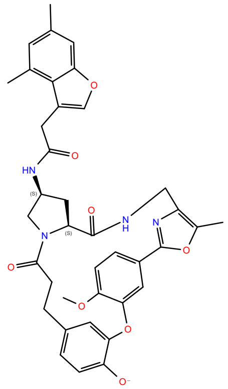

title HIT213842079

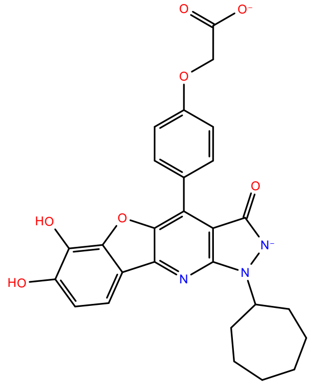

title HIT215011932

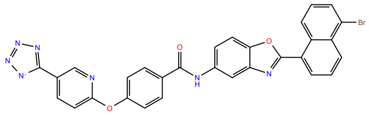

title HIT213718217

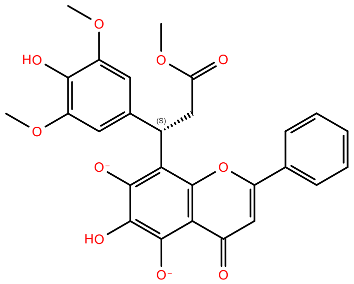

title HIT107251979

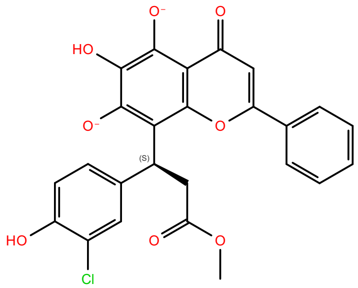

title HIT100470852

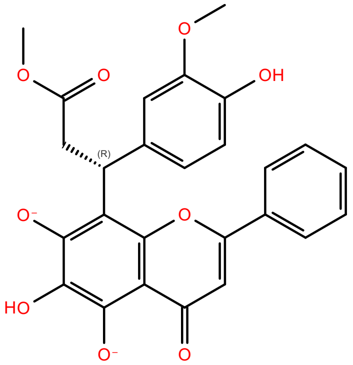

title HIT104388755

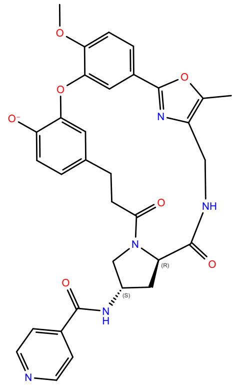

title HIT213843317

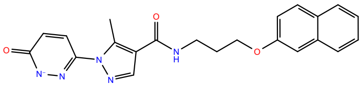

title HIT214835444

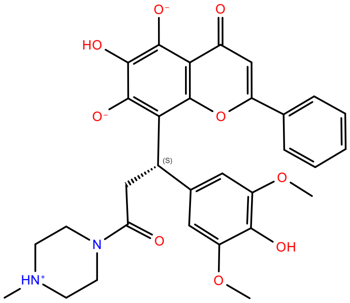

title HIT102215071

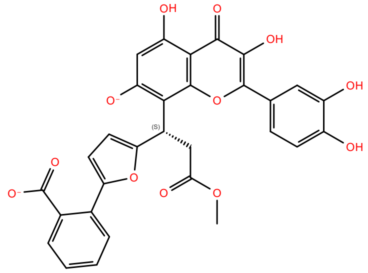

title HIT103063551

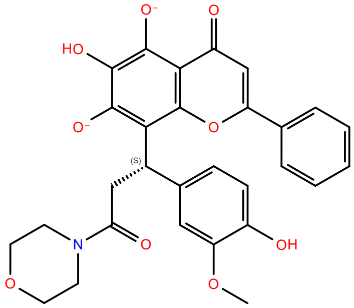

title HIT106546452

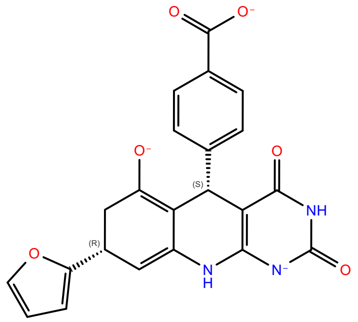

title HIT101149557

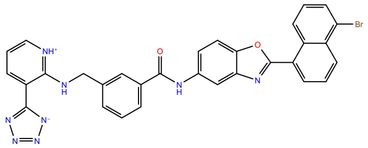

title HIT213717394

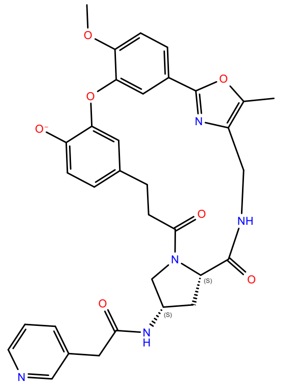

title HIT213843292

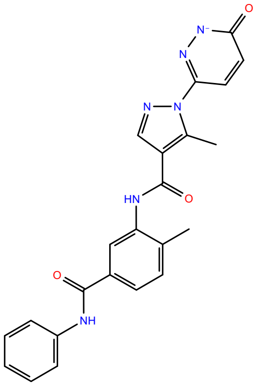

title HIT214755770

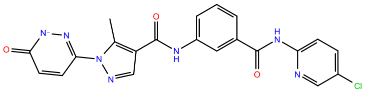

title HIT106834376

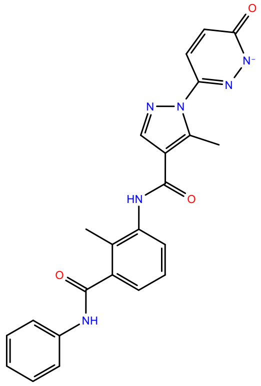

title HIT214813554

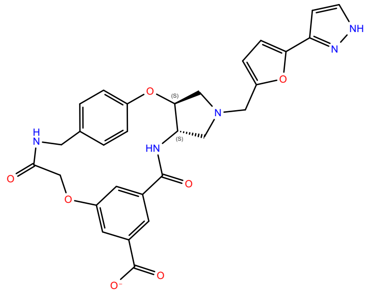

title HIT104201089

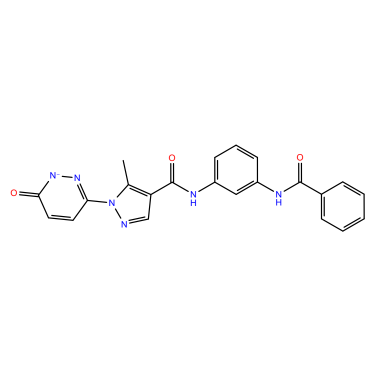
title HIT102002931

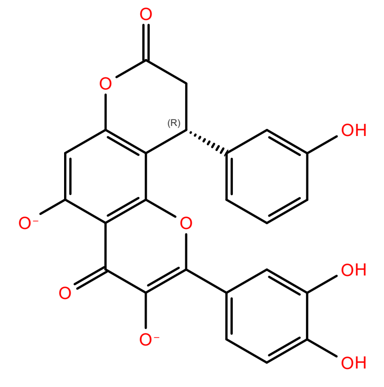
title HIT106938897

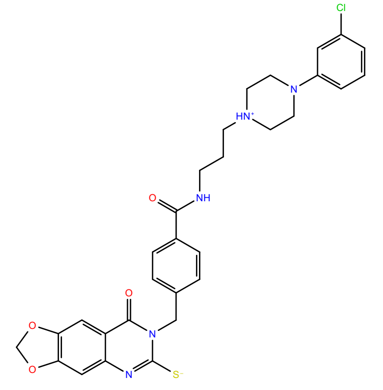
title F3168-2058

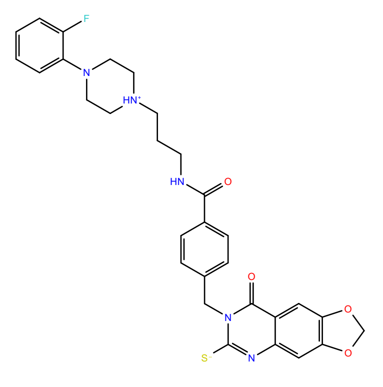
title F3168-2060

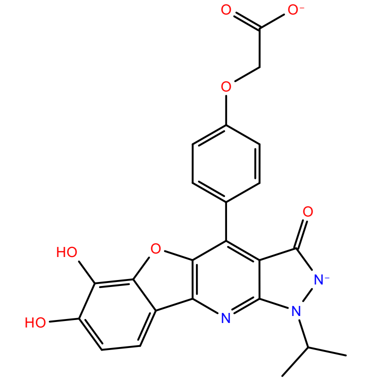
title HIT215015803

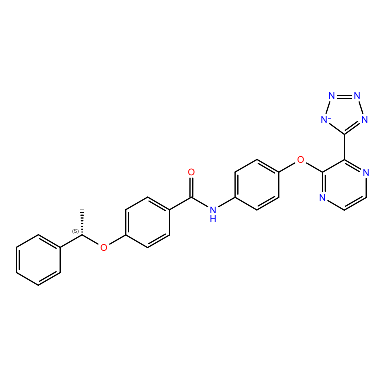
title HIT214660465

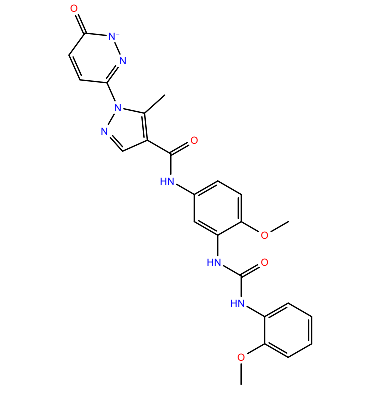
title HIT214787253

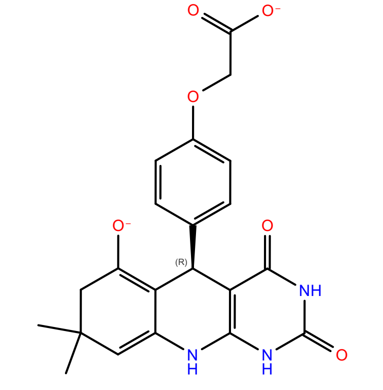
title HIT105180487

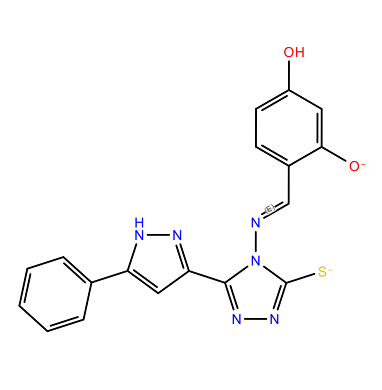
title HIT215001737

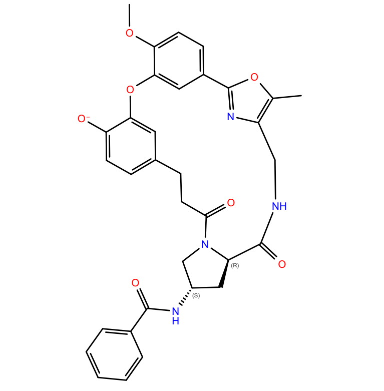
title HIT213843950

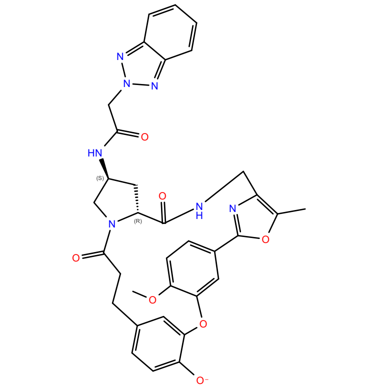
title HIT213842916

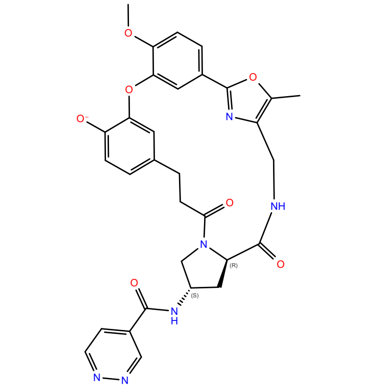
title HIT214021249

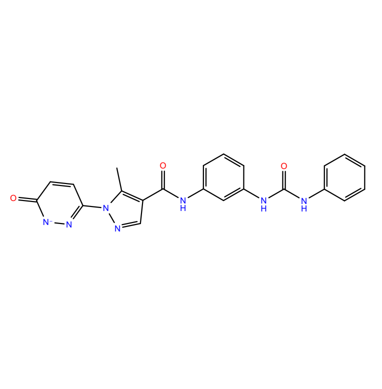
title HIT105005773

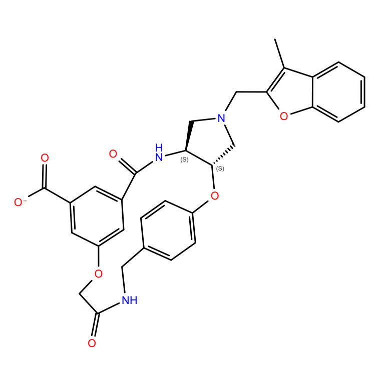
title HIT105918741

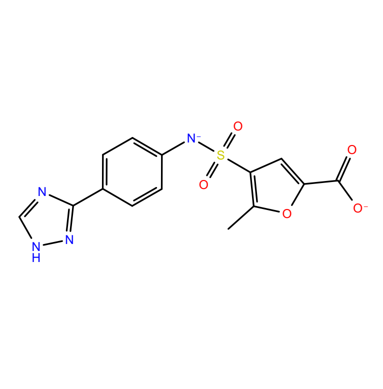
title HIT103236589

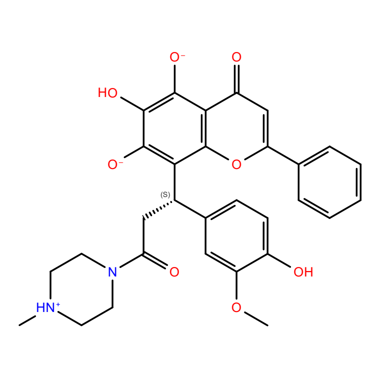
title HIT106034252

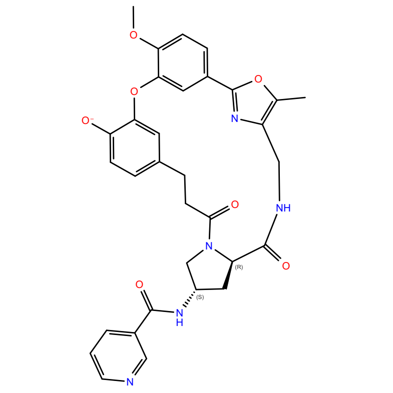

title HIT213843920

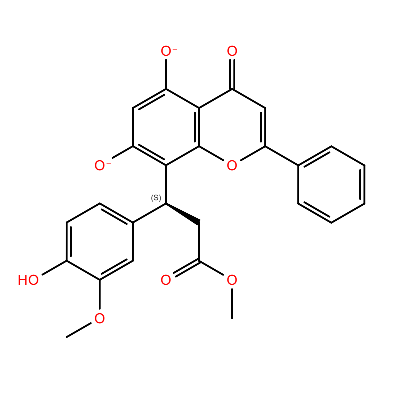

title HIT103512954

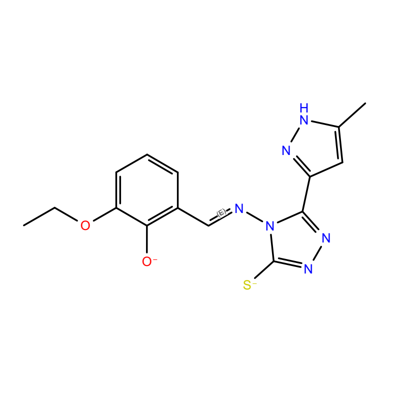

title STOCK5S-24074

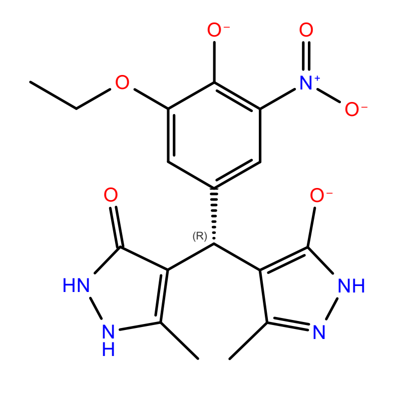

title HIT102702601

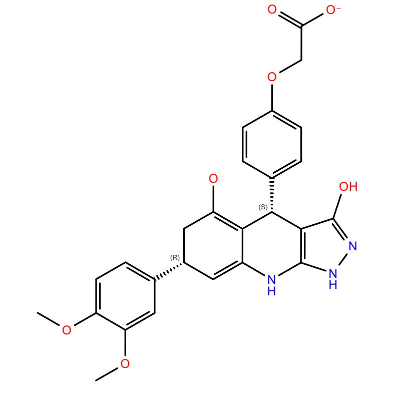

title HIT100081672

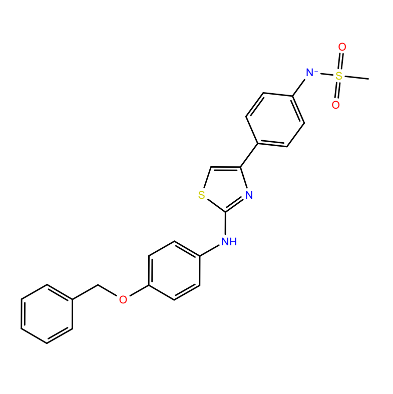

title HIT213545235

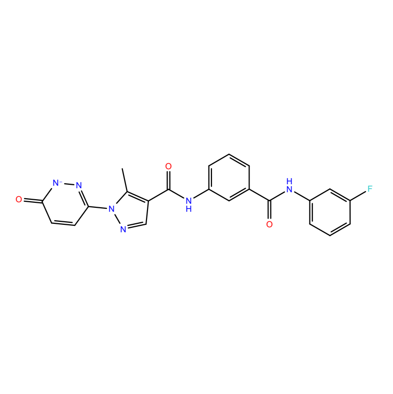

title HIT214757269

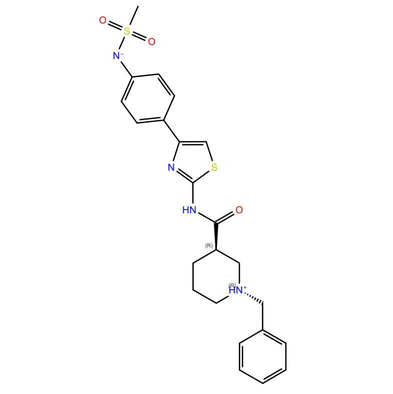

title HIT214762246

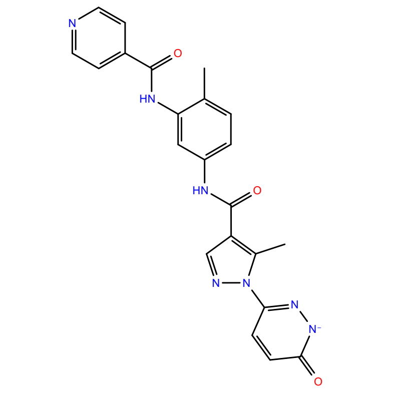

title HIT106071496

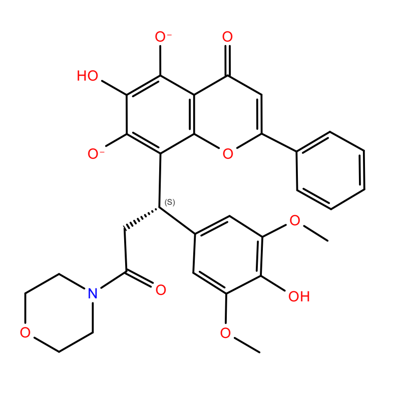

title HIT103276682

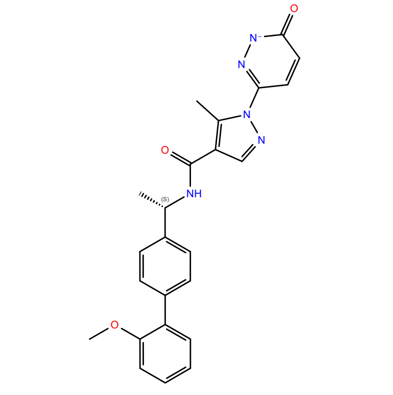

title HIT102269217

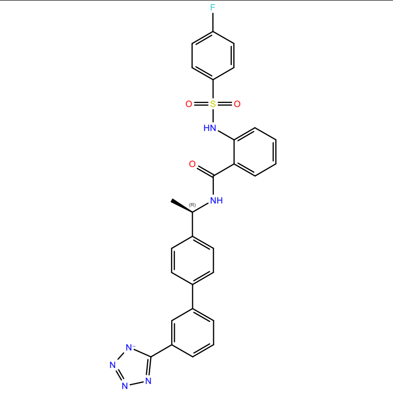

title HIT214679482

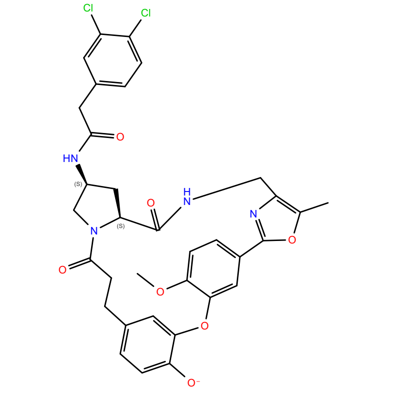

title HIT213843635

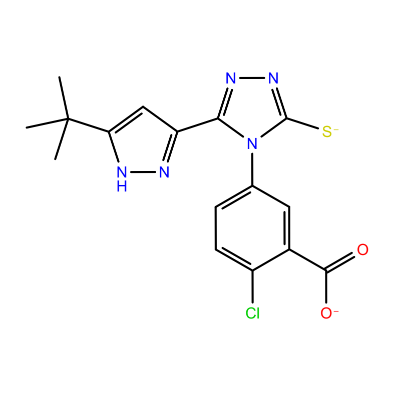

title HIT214427517

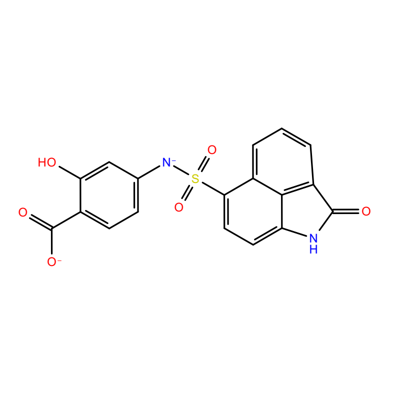

title HIT105801515

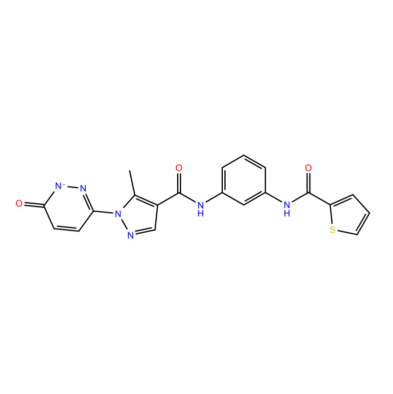

title HIT214811694

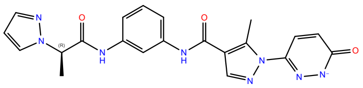

title HIT101041132

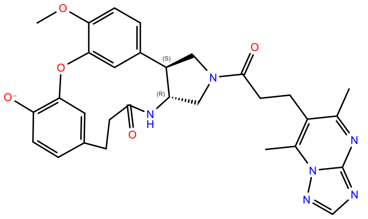

title HIT211916877

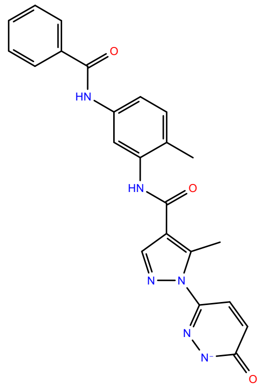

title HIT103574475

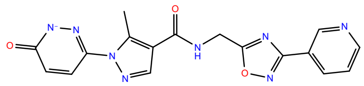

title HIT214761784

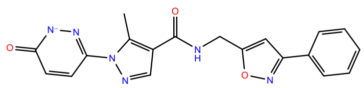

title HIT103212712

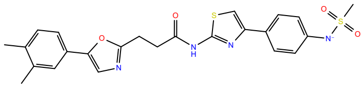

title HIT106658106

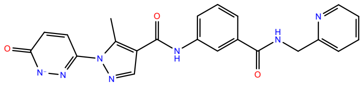

title HIT103499427

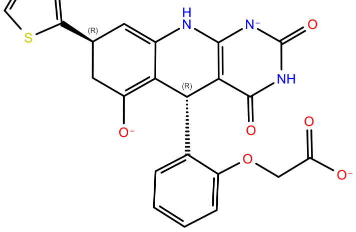

title HIT104389490

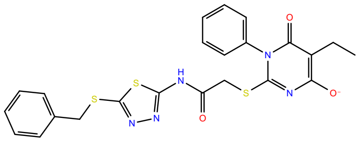

title HIT101393911

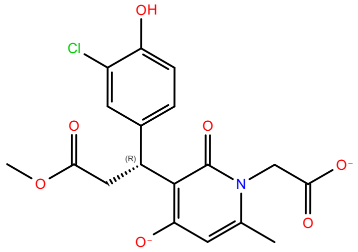

title HIT105786960

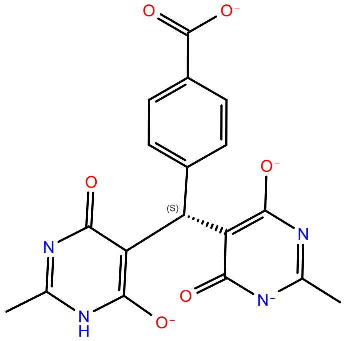

title HIT214416159

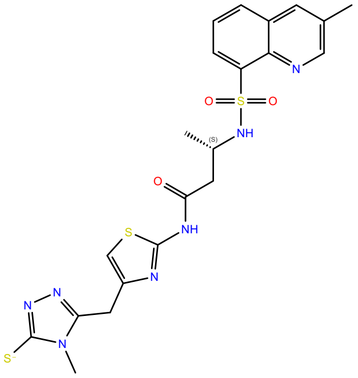

title HIT211668338

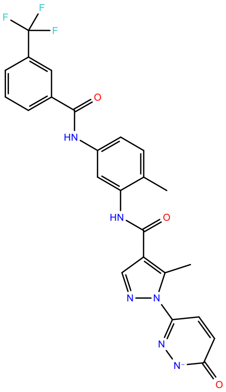

title HIT211320189

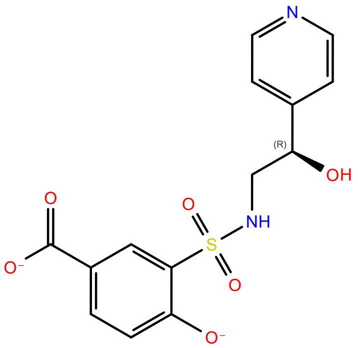

title HIT214686422

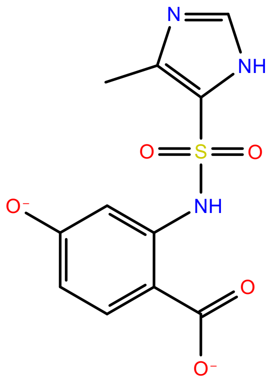

title HIT213745590

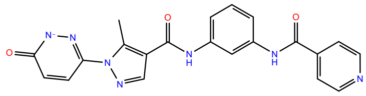

title HIT214815317

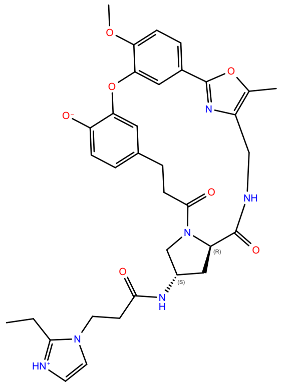

title HIT213843012

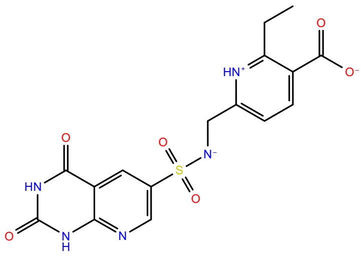

title HIT211574239

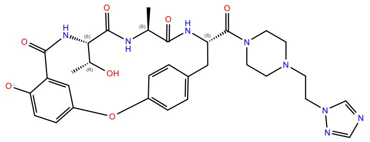

title HIT214969654

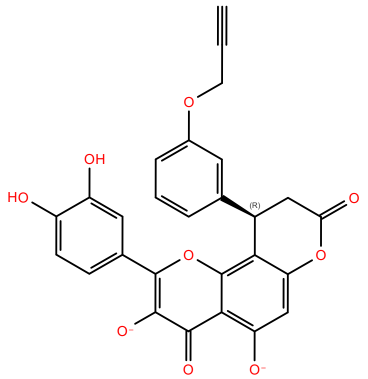

title HIT107136810

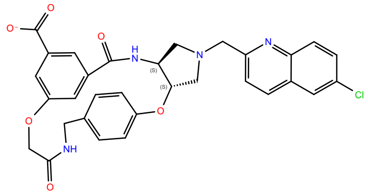

title HIT105115180

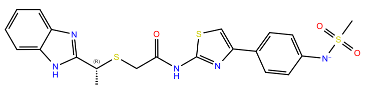

title HIT214799588

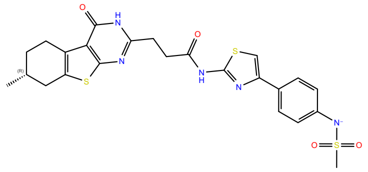

title HIT213729890

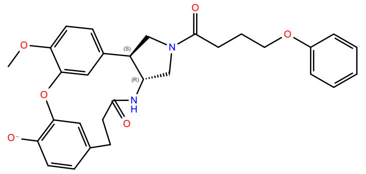

title HIT211916406

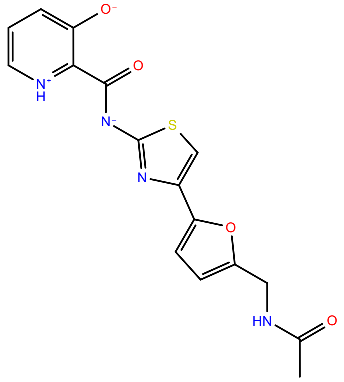

title HIT103990823

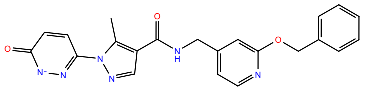

title HIT214754631

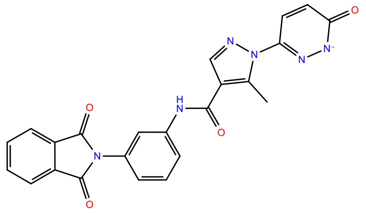

title HIT211544597

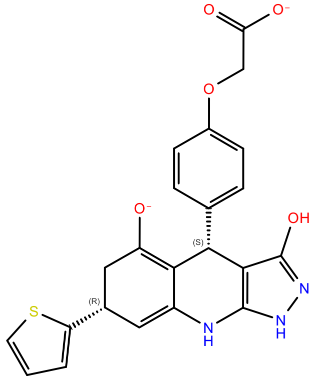

title HIT104026905

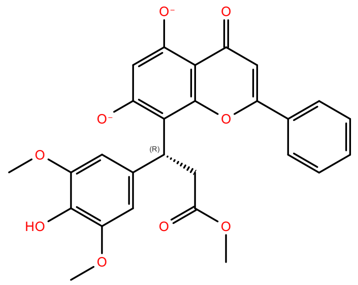

title HIT104500571

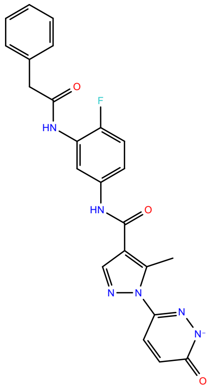

title HIT103373699

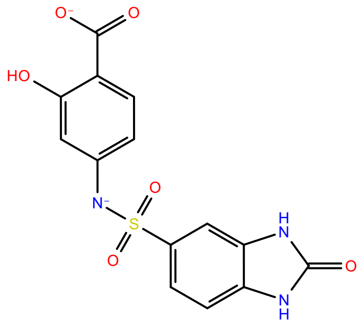

title HIT101130973

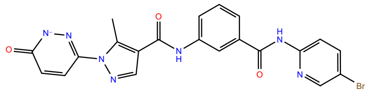

title HIT214915118

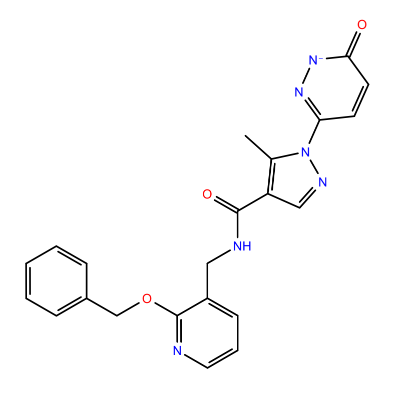

title HIT103516123

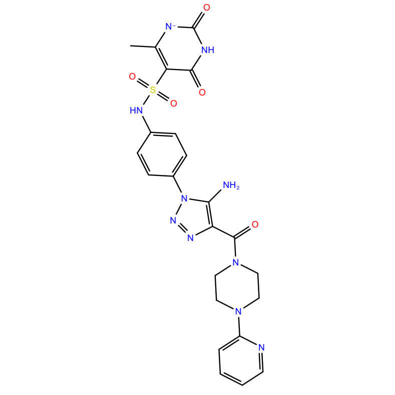

title HIT107056664

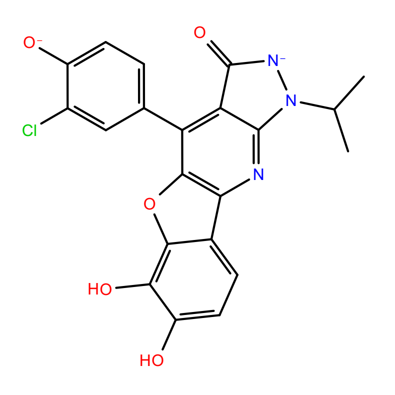

title HIT215008729

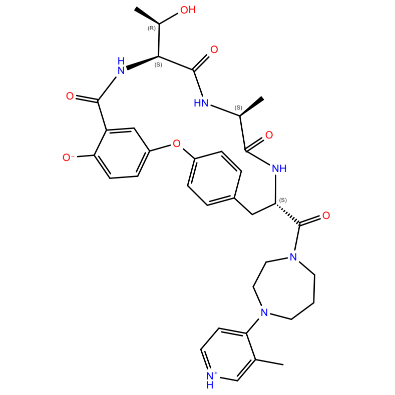

title HIT214980762

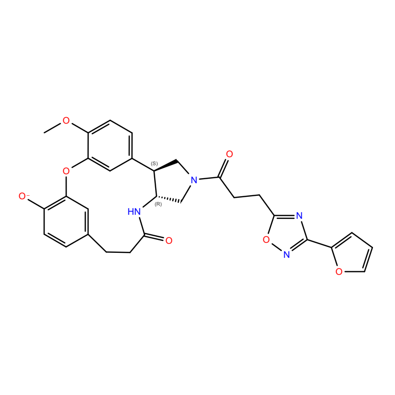

title HIT211917276

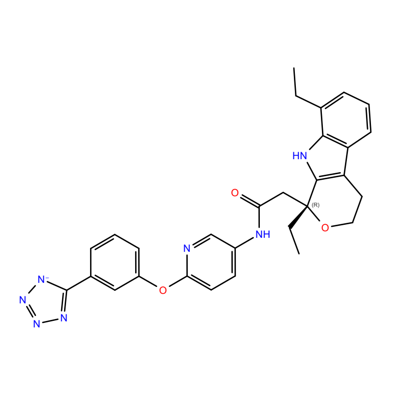

title HIT213730412

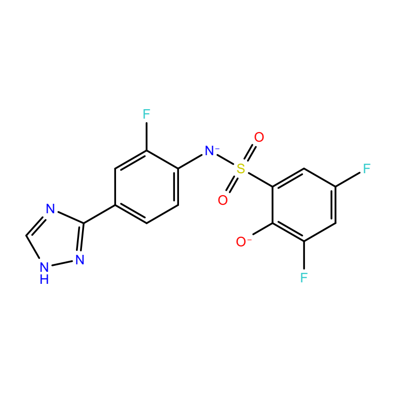

title HIT214008633

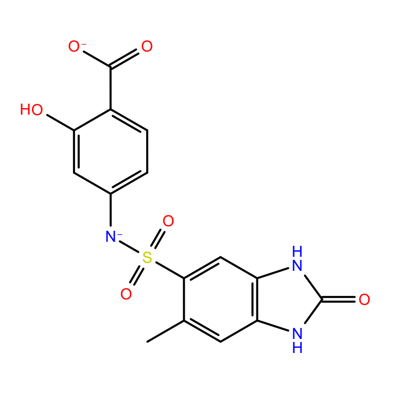

title HIT101509401

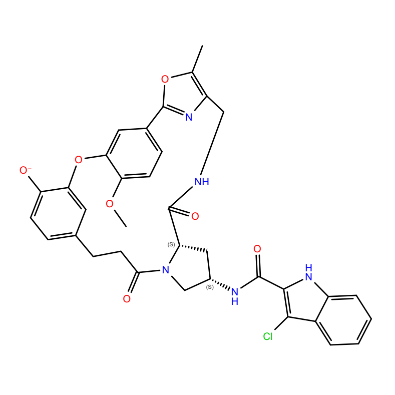

title HIT213843294

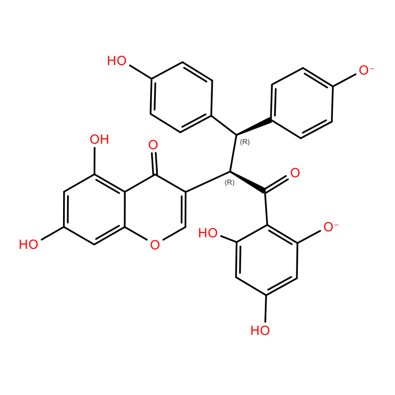

title NP-018776

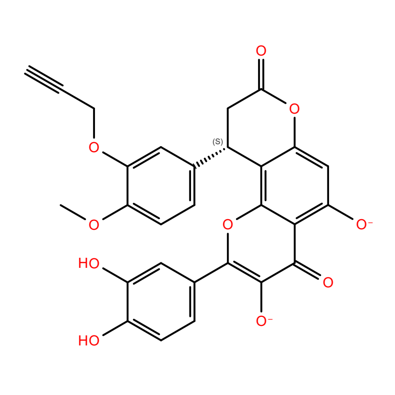

title HIT105270629

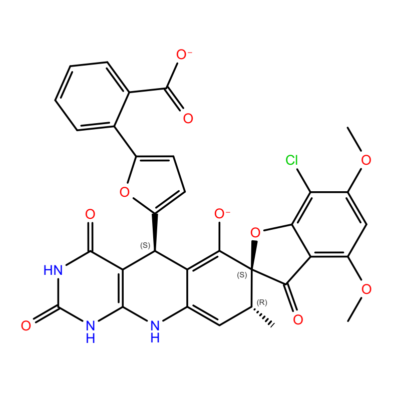

title HIT215015272

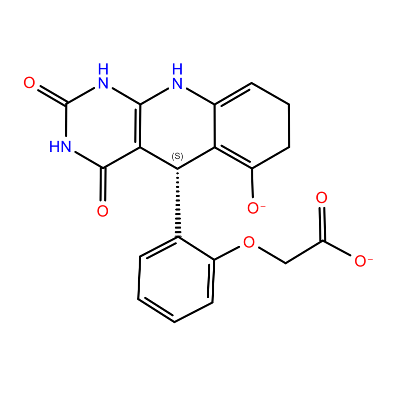

title HIT101675713

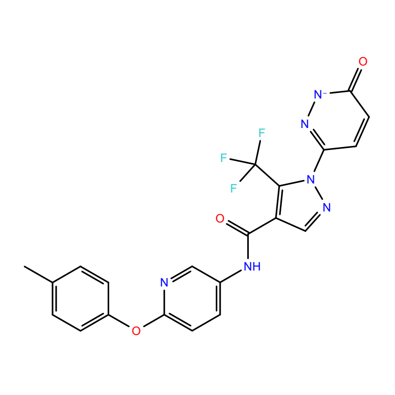

title HIT213638338

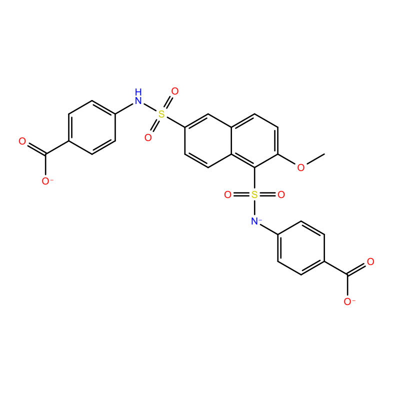

title HIT102268733

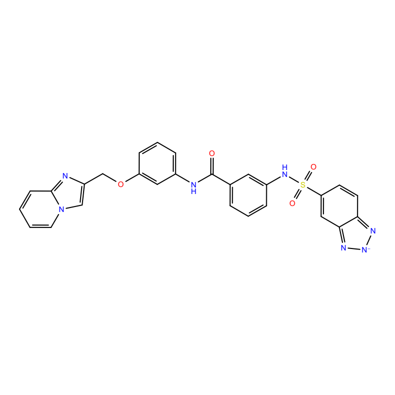

title HIT211670180

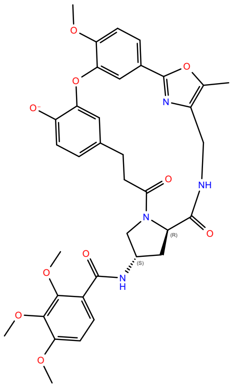

title HIT213842967

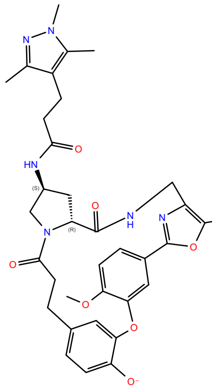

title HIT213843040

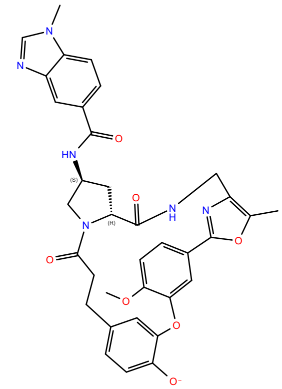

title HIT213845541

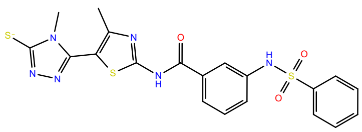

title HIT211551628

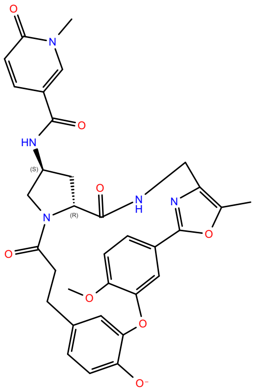

title HIT213842346

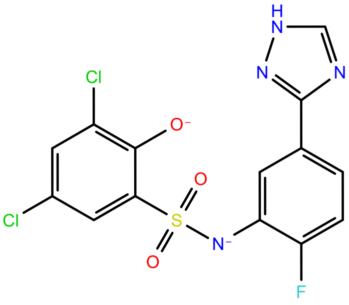

title HIT214704593

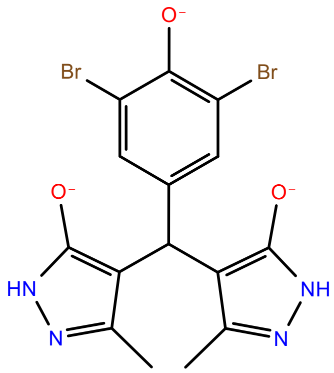

title HIT102968828

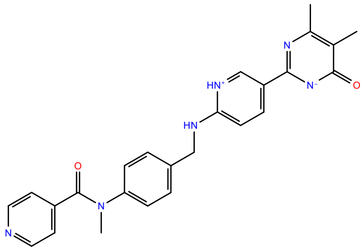

title HIT211567284

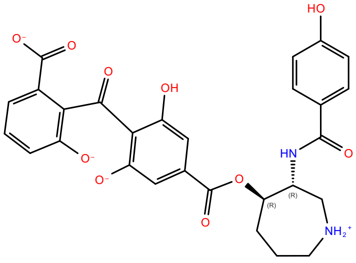

title NP-011482

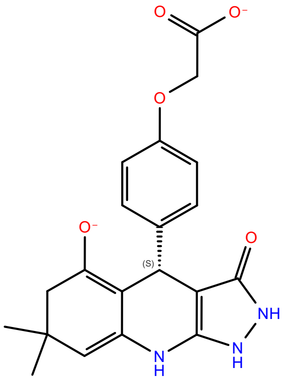

title HIT100759838

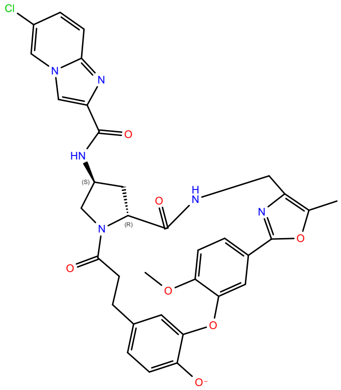

title HIT213842366

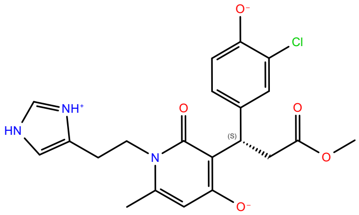

title HIT212573744

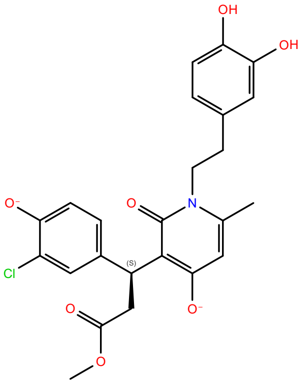

title HIT213019972

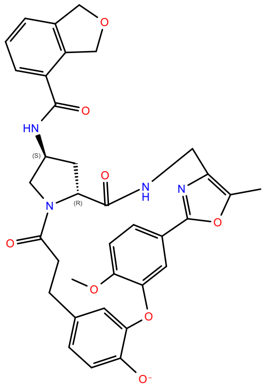

title HIT213845530

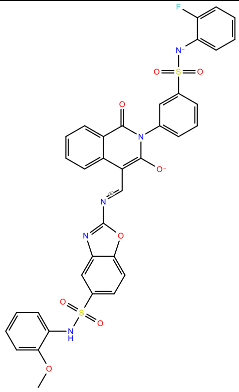

title HIT213726784

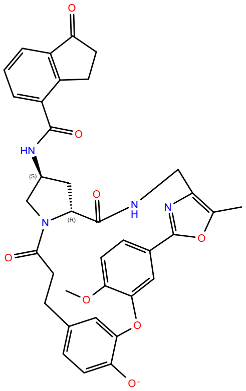

title HIT213845867

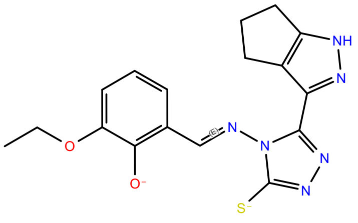

title HIT104302974

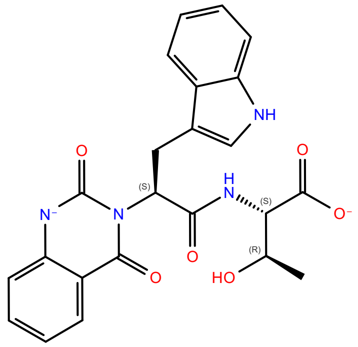

title HIT215013046

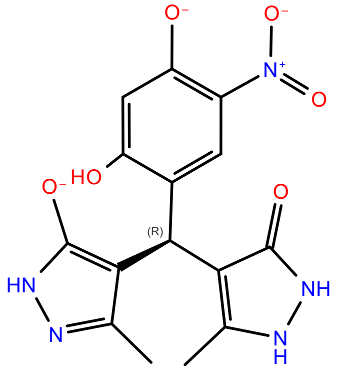

title HIT105471221

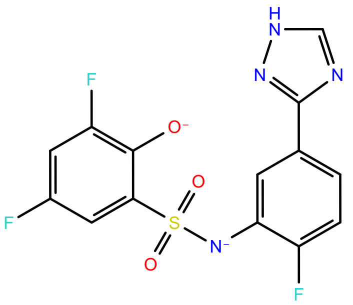

title HIT214008639

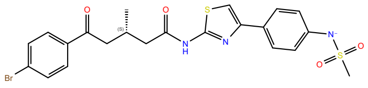

title HIT211669242

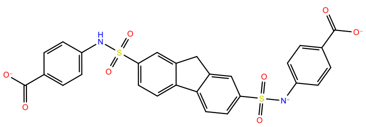

title STOCK7S-6647[illegible]

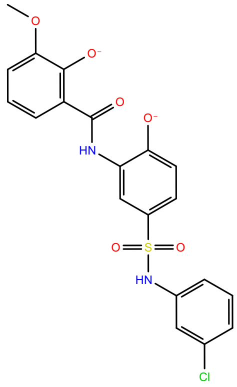

title HIT214567686

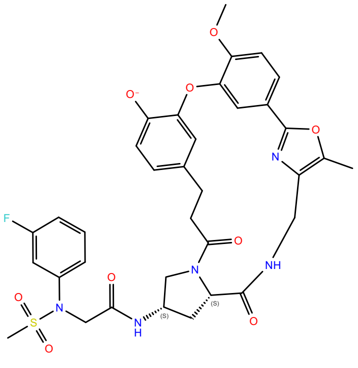

title HIT213846820
